# Most Human Proteins Made in Both Nucleus and Cytoplasm Turn Over within Minutes

**DOI:** 10.1371/journal.pone.0099346

**Published:** 2014-06-09

**Authors:** Sabyasachi Baboo, Bhaskar Bhushan, Haibo Jiang, Chris R. M. Grovenor, Philippe Pierre, Benjamin G. Davis, Peter R. Cook

**Affiliations:** 1 Sir William Dunn School of Pathology, University of Oxford, Oxford, United Kingdom; 2 Chemistry Research Laboratory, Department of Chemistry, University of Oxford, Oxford, United Kingdom; 3 Department of Materials, University of Oxford, Oxford, United Kingdom; 4 Centre d′Immunologie de Marseille-Luminy, Aix-Marseille Université, Marseille, France; 5 Institut National de la Santé et de la Recherche Médicale, U1104, Marseille, France; 6 Centre National de la Recherche Scientifique, Unités Mixtes de Recherche 7280, Marseille, France; The John Curtin School of Medical Research, Australia

## Abstract

In bacteria, protein synthesis can be coupled to transcription, but in eukaryotes it is believed to occur solely in the cytoplasm. Using pulses as short as 5 s, we find that three analogues – L-azidohomoalanine, puromycin (detected after attaching fluors using ‘click’ chemistry or immuno-labeling), and amino acids tagged with ‘heavy’ ^15^N and ^13^C (detected using secondary ion mass spectrometry) – are incorporated into the nucleus and cytoplasm in a process sensitive to translational inhibitors. The nuclear incorporation represents a significant fraction of the total, and labels in both compartments have half-lives of less than a minute; results are consistent with most newly-made peptides being destroyed soon after they are made. As nascent RNA bearing a premature termination codon (detected by fluorescence *in situ* hybridization) is also eliminated by a mechanism sensitive to a translational inhibitor, the nuclear turnover of peptides is probably a by-product of proof-reading the RNA for stop codons (a process known as nonsense-mediated decay). We speculate that the apparently-wasteful turnover of this previously-hidden (‘dark-matter’) world of peptide is involved in regulating protein production.

## Introduction

In prokaryotes, protein synthesis can be coupled to transcription [1]. In eukaryotes, it was debated whether some translation might also be coupled [2], but the discovery of introns seemed to provide the reason why eukaryotes should be different; if nuclear ribosomes translated introns with their many termination codons, too many truncated peptides would be produced, and some of these would be toxic. Clearly, restricting intron-containing RNA to nuclei, and translation to the cytoplasm, prevents such lethal consequences. The debate then fizzled out, but was reignited by the discovery that some nonsense-mediated mRNA decay (NMD) occurs in nuclei [3]. This process involves scanning mRNAs for inappropriately-placed (‘premature’) termination codons (PTCs), and – if found – destruction of the faulty message. As a translating ribosome is the only known mechanism for detecting a termination codon, this places an active ribosome in the nucleus. Despite subsequent reports pointing to some nuclear translation [4]–[8], the consensus remains that proteins are made only in the cytoplasm [9]; most have half lives of many hours [10]–[12], although some turn-over within minutes [13]–[14].

Using pulses as short as 5 s, here we show that three analogues – L-azidohomoalanine, puromycin (detected after attaching fluors using ‘click’ chemistry or immuno-labeling), and amino acids tagged with ‘heavy’ ^15^N and ^13^C (detected using secondary ion mass spectrometry) – are incorporated into both nucleus and cytoplasm in a process sensitive to translational inhibitors. With all three approaches, substantial signal is seen in both compartments. However, our extraordinary finding is that most signal in both nucleus and cytoplasm disappears within minutes. As these structurally-different analogues (detected in different ways) give similar results, it seems that most newly-made peptide – like newly-made RNA [15] – is destroyed almost as soon as it is made.

We then examined what use – if any – the cell might make of this apparently-wasteful turnover. In the case of the nuclear turnover, we tested whether it was involved in NMD. We find that nascent RNA bearing a PTC is eliminated by a mechanism sensitive to a translational inhibitor; this points to an active ribosome proof-reading the nascent RNA prior to its destruction by NMD. [Here we apply the term ‘nascent’ to RNA (and peptide) still associated with the polymerase (or ribosome).] Results are consistent with considerable amounts of translation occurring in nuclei, where a ‘pioneer round of translation’ proofreads the nascent transcript. However, we can only speculate on why so many nascent peptides made in the cytoplasm are degraded.

## Results

### Aha incorporation

**Figure 1:**
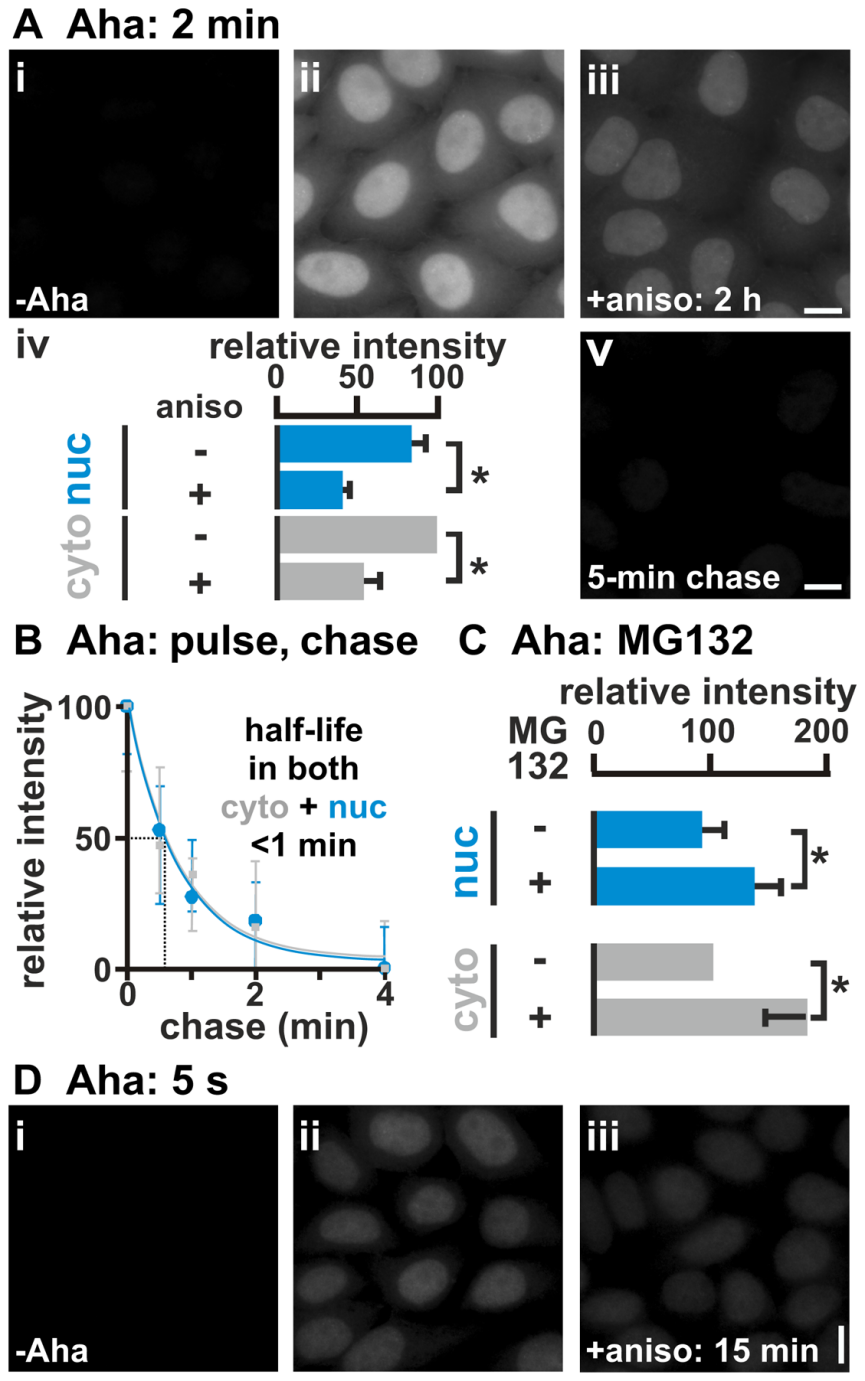
Aha incorporation. HeLa cells were starved of Met (15 and 30 min for 2-min and 5-s pulses, respectively), pulsed ±2 mM Aha, and chased (0–5 min; 0.2 mM Met without Aha). After fixation and ‘clicking’ on Alexa555, DNA was counterstained with DAPI, images collected using a wide-field microscope, and fluorescence intensities (± SD) in the cytoplasm (*cyto*) and nucleus (*nuc*) normalized relative to values in the untreated cytoplasm. *: *P*<0.0001 (Student's two-tailed *t* test, *n* = 20 cells). Bars: 10 µm. (**A**) 2-min Aha pulse. (i,ii) Aha labels both nucleus and cytoplasm, with the nucleus being the brightest. (iii) Pretreatment with anisomycin (aniso; 100 µg/ml; 2 h) reduces signals in both nucleus and cytoplasm. (iv) Slightly more signal is found in the larger area of the cytoplasm. (v) Signals in both compartments disappear during a chase. (**B**) After a 2-min pulse, signals in both compartments disappear quickly during a chase. Best fits of simple exponential curves to the data are included, but note that we do not know how many kinetic populations there might be (Materials and Methods S1). (**C**) MG132 (100 µg/ml; 2 h) increases signal given by a 2-min pulse. (**D**) 5-s Aha pulse. (i-iii) Signal is sensitive to anisomycin (aniso; 100 µg/ml; 15 min).

Aha is an analogue of methionine (Met), an essential amino acid, and it is incorporated into proteins both at the N terminus and internally; as it possesses a reactive azide group, ‘click’ chemistry is widely used to conjugate alkyne-containing fluors on to Aha-containing peptides, before localization of those peptides [16]–[17]. All reports indicate that Aha-bearing peptides behave much like their Met-containing counterparts; for example, zebrafish larvae develop normally when grown in Met-free and Aha-containing medium for two days [18]. [However, Aha is not incorporated as efficiently as Met by the bacterial Met-tRNA synthetase [19].] When HeLa cells are starved of Met to deplete endogenous pools, grown in Aha for 2 min, fixed, and Alexa555 ‘clicked’ on to incorporated Aha, fluorescence is seen in both cytoplasm and nucleus (**Fig. 1A** **ii**). Pre-incubation with the translational inhibitor, anisomycin [20], reduces Aha incorporation (**Fig. 1A** **iii**; see also the legend to **Fig. S1**). [The anisomycin concentration and pre-incubation time applied here are routinely used to inhibit translation to the levels we see [21]; shorter pre-incubation times of 15–30 min will be used in critical experiments, and other inhibitors (e.g., puromycin, cycloheximide) give similar results (**Fig. S1A**, legend).] Although signal is brightest in nuclei, quantitative analysis shows that slightly more is found in the larger area of the cytoplasm (**Fig. 1A** **iv**). Since starvation stresses cells and this might have unforeseen results, the experiment was repeated without prior starvation; although signals are now fainter, again nuclei are the brightest and integrated signal over the cytoplasm is the highest (**Fig. S1A**). Similar results are obtained with primary (diploid) human umbilical vein endothelial cells (HUVECs; **Fig. S1A**), so results are not peculiar to a transformed cell like HeLa.

A pulse-chase experiment confirms that Aha is incorporated by a metabolic process, and that newly-made peptides are degraded rapidly. After a 2-min Aha pulse, and regrowth in Met for up to 5 min; nuclear and cytoplasmic signals fall with half-lives of <1 min at 37°C (**Fig. 1A** **v** and **Fig. 1B**) – but not at 4°C (**Fig. S1B**). Pre-treatment with the proteasomal inhibitor, MG132 [22], increases nuclear and cytoplasmic fluorescence (**Fig. 1C**). Essentially no signal remains after a 5-min chase (**Fig. 1A** **v**). This contrasts with the known half-lives of human proteins (measured using pulses lasting days) of ∼20 h (the range of half-lives covers minutes to many tens of hours; [11]–[12]). However, a 60-min pulse gives sufficient incorporation to allow some signal to survive a 5-min chase (**Fig. S1C**). These results are consistent with most newly-made Aha-tagged peptides being destroyed within minutes by the proteasome (so inhibiting it increases signal), and with a tiny fraction surviving to contribute to the ‘mature’ proteome (so only the latter is detected using long pulses). Of course, this does not exclude the possibility that other proteases contribute to the turnover [23]. In the unlikely event that Aha is incorporated by some unknown non-ribosomal mechanism, the resulting product must then be degraded by the proteasome. This makes it unlikely that the signal could just be due to incorporation of Aha into an amino-acyl tRNA.

A ribosome polymerizes ∼5 amino acids per second *in vivo* [24], so a typical human protein with ∼400 residues is polymerized in ∼80 s. Therefore, the nuclear signal could result from cytoplasmic synthesis followed by rapid nuclear import. The use of a 5-s pulse eliminates this possibility, as signals are still seen in both compartments (**Fig. 1D**, **Fig. S1D**). Since only one in sixteen proteins (i.e., 400/[5×5]) will be completed during this pulse, most (>90%) nuclear signal must arise from peptides made in nuclei.

To confirm that the signal seen during a 5-s pulse did not result just from aminoacylation of tRNA (rather than a subsequent incorporation by a ribosome into peptide), we also examined whether destruction of RNA reduced the signal. In contrast to such an expectation, treatment with a cocktail of RNases (after fixation and immediately prior to ‘clicking’) increased the relative intensity in nucleus and cytoplasm to 189±26% and 139±22%, respectively (**Fig. S1**, legend). This is consistent with RNase-sensitive material in both nucleus and cytoplasm (i.e., RNA) preventing access of the ‘click’ reagent to Aha incorporated into RNA-free complexes (i.e., peptide) in both compartments. [See the legend of **Figure S1** for an analogous control involving DNase that demonstrates that Aha is likely to be incorporated into DNA-free complexes.]

### Puromycin incorporation

**Figure 2:**
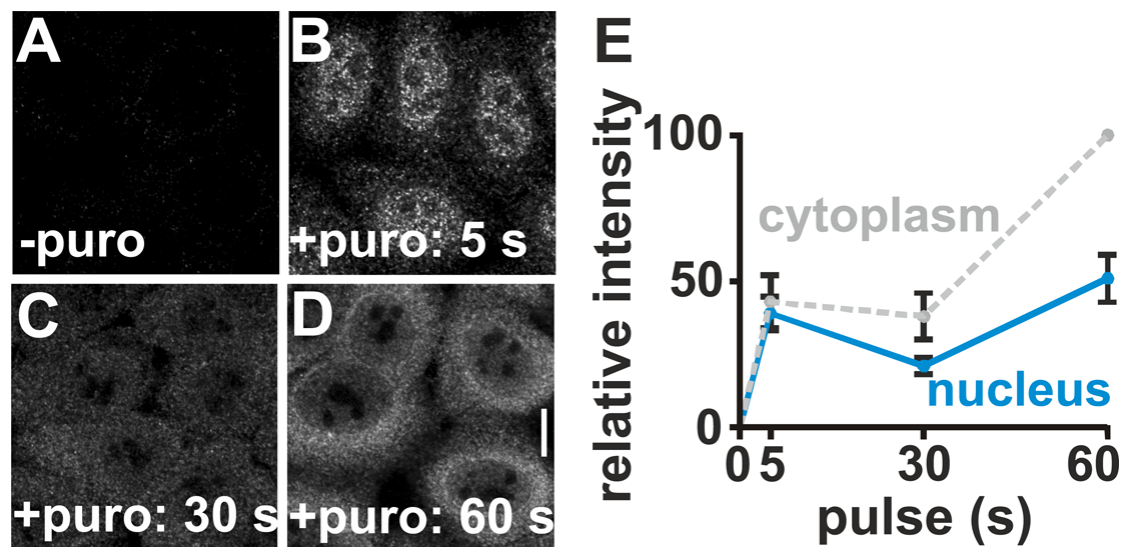
Puromycin incorporation. HeLa cells were pre-treated with cycloheximide (100 µg/ml; 15 min) to slow ribosomes, pulsed with puromycin (puro; 91 µM; 0–60 s), fixed, puromycylated peptides immuno-labeled with Cy3, DNA stained with DAPI, and images collected. (**A-D**) Typical confocal sections through the centre of nuclei. A 5-s pulse gives bright nuclear foci. After 30 s, cytoplasmic and nuclear signals are more similar and diffuse. After 60 s, the peri-nuclear region is the brightest. Bar: 10 µm. (**E**) Using wide-field images, Cy3 intensities (±SD; *n* = 20 cells) are expressed relative to the cytoplasmic value after a 60-s pulse.

Here we modify an approach applied previously to show that some translation occurs in nuclei [5]; however, we use shorter pulses and unpermeabilized cells. Cells are first treated with cycoheximide to ‘freeze’ ribosomes, and then incubated with puromycin – a structural mimic of aminoacyl-tRNA which is incorporated into the C-termini of nascent peptides; finally, the puromycylated and still-nascent peptides are immuno-localized using an anti-puromycin antibody [5], [25]. After growth in cycloheximide for 15 min and puromycin for 5 s, puromycylated peptides are seen in bright nucleoplasmic foci in ‘confocal’ images (**Fig. 2A,B**; **Fig. S2A** compares ‘wide-field’ and ‘confocal’ images). After 30 s in puromycin, the nuclear signal decreases and becomes more diffuse (**Fig. 2C**). After 60 s, the peri-nuclear region becomes the brightest (**Fig. 2D,E**). These changes mimic those seen previously and are simply interpreted in light of the known behaviour of puromycylated peptides: after dissociating from ribosomes, many accumulate at exit sites on the smooth endoplasmic reticulum (SER) before passing through the Golgi apparatus to the exterior [5], [25]–[27]. Additional results obtained using short pulses and the longer ones applied by David et al. [5] are also consistent with this interpretation (**Fig. S3A,B**). Moreover, similar results are obtained when pre-incubation with cycloheximide is omitted, so the labeling again cannot result from some unknown stress-induced response (**Fig. S3C**). If nuclear translation is coupled to transcription [4], inhibiting transcription should reduce nuclear (but not cytoplasmic) incorporation, and it does (**Fig. S3D**).

### NanoSIMS

**Figure 3:**
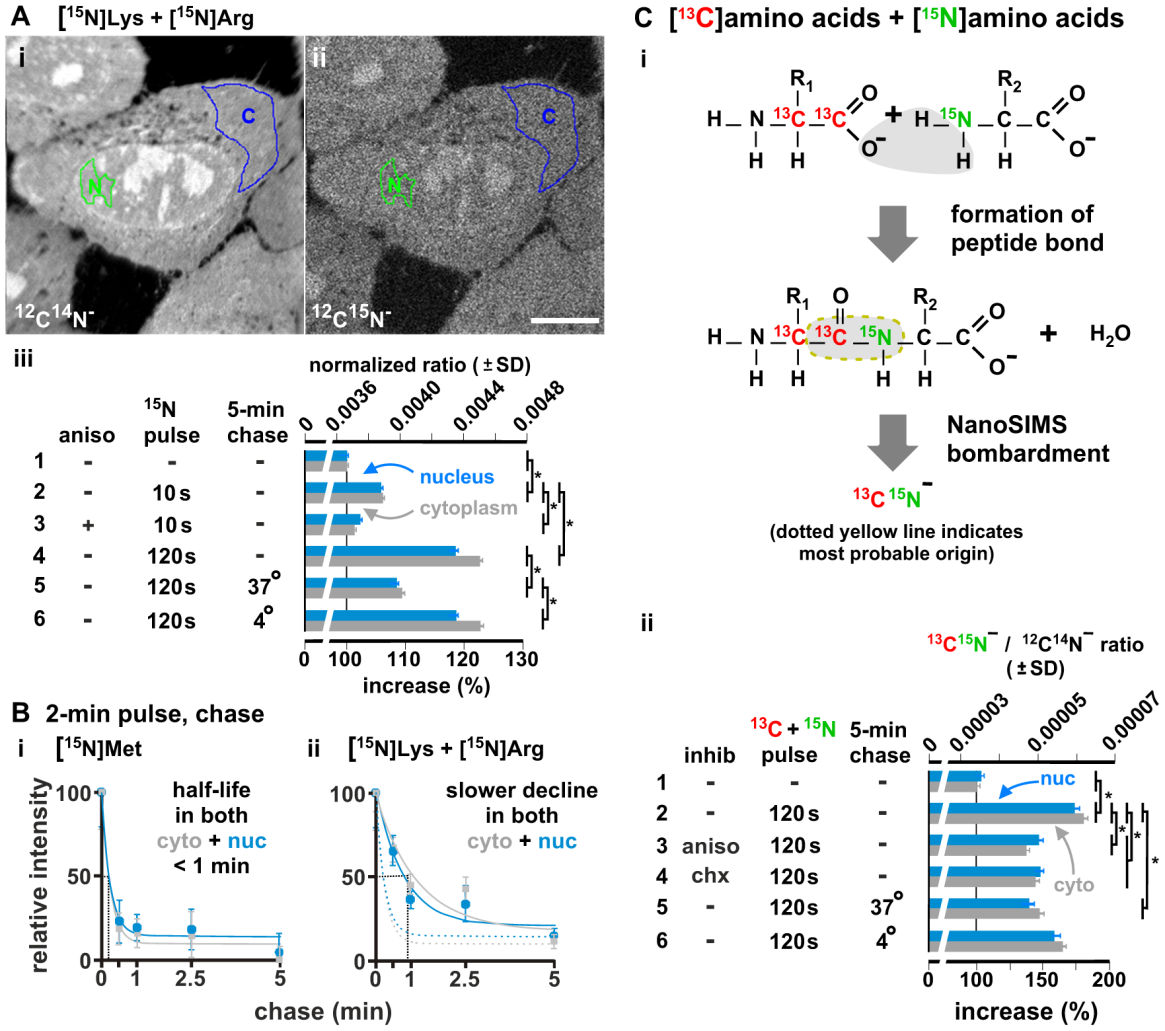
Incorporation of heavy amino acids detected using NanoSIMS. HeLa were starved of relevant amino acid(s), grown (10, 120 s) in heavy amino acids, fixed, sectioned, images collected, and distributions of light (^12^C^14^N^−^), heavy (^12^C^15^N^−^), or heavy-heavy ions (^13^C^15^N^−^) measured. In some cases, inhibitors were present during starvation and the pulse, or chases followed the pulse. (A) An example after 30-min starvation of Lys + Arg, and 2-min growth in 8 mM [^15^N]Lys +4 mM [^15^N]Arg. (i,ii) Images show distributions of light and heavy ions in one section (regions of interest in the nucleus, N, and cytoplasm, C, indicated). Bar: 10 µm. (iii) Signals due to heavy ions are expressed as ratios (± SD) relative to total numbers of ions (i.e., ^12^C^15^N^−^/[^12^C^15^N^−^+^12^C^14^N^−^]) normalized to the natural abundance of ^15^N. *: *P*<0.0001 (Student's two-tailed t test; *n* = *19–26* cells). *Row 1*: cells unexposed to the heavy labels give a ratio characteristic of the natural abundance. *Rows 2–3*: a 10-s pulse in heavy medium increases ratios, and anisomycin (aniso; 100 µg/ml; 30 min) reduces this. *Rows 4–6*: a 120-s pulse yields higher ratios, which are reduced by a chase at 37°C (but not 4°C). (B) After 30-min starvation in the absence of either Met (or Lys + Arg), and a 2-min pulse in 2 mM [^15^N]Met (or 8 mM [^15^N]Lys +4 mM [^15^N]Arg), signals in both compartments decline quickly during a chase. Best fits of simple exponential curves to the data are included, but note that we do not know how many kinetic populations there might be (Materials and Methods S1). (i) With [^15^N]Met (which is incorporated into the N-terminus and internally), essentially all label disappears by 5 min. (ii) After labeling with [^15^N]Lys + [^15^N]Arg (which are never incorporated into the N-terminus), the decline is slower (which is consistent with these internal labels being preferentially incorporated into a minor, longer-lived, fraction). For comparison, curves (dotted lines) are reproduced from panel (i). (C) Signal is due to the formation of peptide bonds. Cells were starved (15 min) of all amino acids, and grown (2 min) in [^13^C]amino acids + [^15^N]amino acids ±100 µg/ml anisomycin or 100 µg/ml cycloheximide. (i) Some peptide bonds are then formed by a ribosome covalently linking a carboxyl ^13^C atom in one [^13^C]amino acid to an amino ^15^N atom in a [^15^N]amino acid; the NanoSIMS bombardment generates (directly or indirectly) ^13^C^15^N^−^. R_1_ and R_2_: different residues. (ii) After collecting images, distributions of ^13^C^15^N^−^ and ^12^C^14^N^−^ ions in regions of interest in the nucleus (nuc) and cytoplasm (cyto) were measured; signals due to (heavy-heavy) ^13^C^15^N^−^ are expressed as ratios (± SD) relative to those due to (light-light) ^12^C^14^N^−^. *: *P*<0.0001 (Student's two-tailed t test; *n* = 11–18 cells). *Row 1*: cells unexposed to heavy labels give ratios characteristic of the natural abundance. *Row 2*: growth in both heavy labels increases ratios. *Rows 3,4*: 100 µg/ml anisomycin (aniso) or cycloheximide (chx) reduces ratios. *Rows 5,6*: a chase at 37°C also reduces ratios, whilst one at 4°C gives less reduction.

NanoSIMS (high-resolution secondary-ion mass spectrometry [28]) involves raster-scanning the surface of a fixed specimen with a focused ion beam to vaporize the surface of the specimen, using a mass spectrometer to measure the mass/charge ratio of secondary ions ejected during the bombardment, and then creating an image that reflects the isotopic composition of the surface. It combines high sensitivity and mass resolution, with a spatial resolution of ∼100 nm in the *x* and *y* axes, and a few nanometers in the *z* axis. HeLa were grown for 2 min in ‘heavy’ [^15^N]Lys and [^15^N]Arg, and **Figure 3A** **i** illustrates an image depicting the distribution of (‘light’) ^12^C^14^N^−^ ions in a typical section through the middle of a nucleus (prepared as for conventional electron microscopy). Signal due to (‘heavy’) ^12^C^15^N^−^ ions is lower (**Fig. 3A** **ii**); it contains contributions from ^15^N naturally present in the biosphere, plus some from the tagged amino acids. To compare levels in different samples, signal due to heavy ^12^C^15^N^−^ ions is expressed as a ratio relative to both heavy and light ions, and normalized relative to the natural abundance of ^15^N (**Fig. 3A** **iii**). Then, a control unexposed to heavy medium yields a ratio characteristic of the natural abundance (*row 1*). A 10-s pulse in heavy medium increases ratios by ∼6% (*row 2*); anisomycin reduces this increase (*row 3*). A 120-s pulse yields still higher ratios (*row 4*) – which are reduced by a chase at 37°C (*row 5*), but not 4°C (*row 6*). These results confirm that some peptides are made in both nucleus and cytoplasm, and that they turn over rapidly.

We next assessed how rapidly peptides made during a 2-min pulse with either [^15^N]Met (which is incorporated by the ribosome into both the amino terminus and internally), or [^15^N]Lys plus [^15^N]Arg (which are not incorporated by the ribosome at the amino terminus). Most peptides tagged with [^15^N]Met disappear from both nucleus and cytoplasm within several tens of seconds (**Fig. 3B** **i**). Peptides tagged with [^15^N]Lys plus [^15^N]Arg disappear slightly more slowly (**Fig 3B** **ii**). These results are consistent with a scenario in which most translating ribosomes abort soon after initiation (with rapid destruction of the resulting peptides), and with only a minority going on to generate longer peptides that contribute to the ‘mature proteome’. Then, during a pulse with an end-biased label like [^15^N]Met, most label is incorporated into short (aborting) peptides that turn over quickly. But after a pulse with labels that can only be incorporated by ribosomal polymerization into the middle of a peptide (i.e., [^15^N]Lys, [^15^N]Arg), a larger minority is incorporated into longer peptides that turn over more slowly.

We further confirmed that much of the signal due to heavy label results from the formation of peptide bonds – the covalent linkage of a (carboxyl) carbon in one amino acid with the (amino) nitrogen in another. HeLa cells were starved of all amino acids for 15 min, and then incubated for 2 min in both [^13^C]amino acids and [^15^N]amino acids (each set being uniformly-labeled with the respective heavy atom). Protein synthesis will covalently link some ^13^C atoms with ^15^N atoms; this should then yield (heavy-heavy) ^13^C^15^N^−^ ions during NanoSIMS [29] (**Fig. 3C** **i**). Most ions of this type arise from incomplete fragmentation of one peptide, or indirectly by complete fragmentation of one peptide to monatomic ^13^C and ^15^N which then recombine to give the diatomic ion; a minor fraction is derived by complete fragmentation of two different molecules followed by recombination of ^13^C from one with ^15^N from the other [29]. Then, detection of heavy-heavy ions at levels above those found naturally in the biosphere should primarily reflect the presence of ^13^C and ^15^N atoms lying close together within the same molecule in the sample.

To compare levels in different samples, signal due to the (heavy-heavy) ^13^C^15^N^−^ ions is expressed as a ratio relative to their (light-light) ^12^C^14^N^−^ counterparts (**Fig. 3C** **ii**). Then, a control unexposed to heavy medium yields the ratio characteristic of the sum of the natural abundance of both heavy isotopes (*row 1*). A 120-s pulse in heavy-heavy medium increases ratios in both nucleus and cytoplasm (*row 2*). The scale of this increase is reduced by two ribosomal inhibitors (anisomycin and cycloheximide; *rows 3,4*), and by a chase at 37°C (*row 5*) – whilst a chase at 4°C has less effect (*row 6*). These results are consistent with a significant fraction of the ^13^C^15^N^−^signal resulting from the formation of new peptide bonds in both nucleus and cytoplasm.

### A possible role for the nuclear turnover in proof-reading nascent RNA

**Figure 4:**
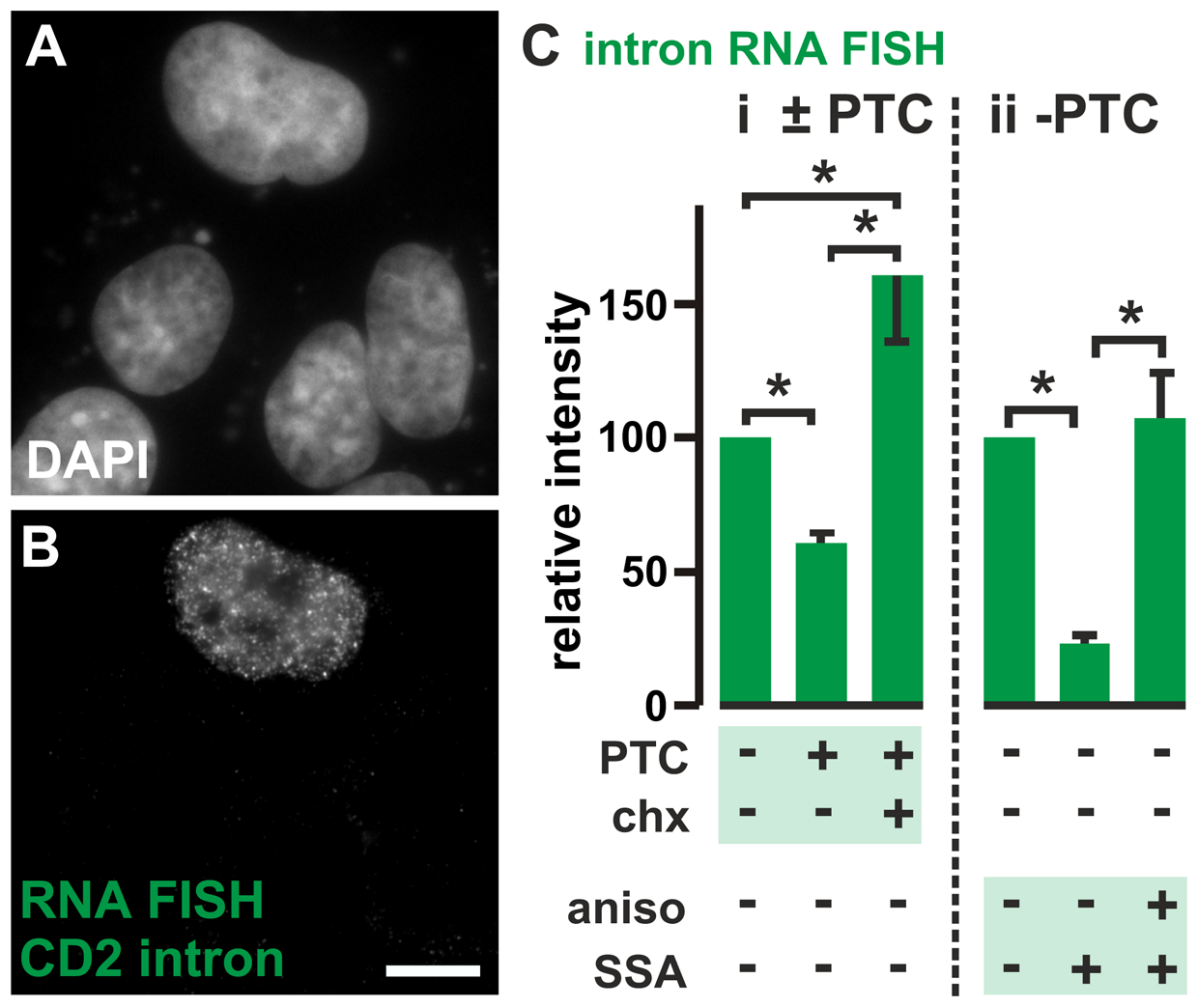
Effects of PTCs on nascent *Cd2* RNA. Cos-7 cells (which encode the SV40 T antigen) were co-transfected with constructs encoding the ‘Tet’ repressor and a test vector (with the ‘Tet’ promoter driving *Cd2* ± a PTC). By 24(which also encodes the SV40 *ori*) replicates to give ∼8,000 mini-chromosomes/cell; the ‘Tet’ promoter is silent and no *Cd2* RNA is detected. Now doxycycline (10 µM; 45 min) is added, cells fixed 26 h after transfection, intronic *Cd2* RNA detected by RNA FISH, DNA stained with DAPI, and images collected using a wide-field microscope. (**A,B**) Two views of one field after co-transfecting the PTC^–^ vector (only the cell at the top was transfected and expresses intronic *Cd2* RNA in nuclear foci). Bar: 10 µm. (**C**) After subtracting background, intensities (± SD) seen in nuclei are expressed relative to the value found in untreated cells transfected with the PTC¯ vector. *: *P*<0.0004 (Student's two-tailed *t* test, *n* = 20 cells). (**i**) A PTC reduces levels of intronic RNA, but cycloheximide (chx; 100 µg/ml; 2 h) more than reverses the effect. (**ii**) After transfecting the PTC¯ vector, SSA (100 ng/ml; 45 min) reduces levels of intronic RNA, and anisomycin (aniso; 100 µg/ml; 2 h) reverses this effect.

Finally, we examined what use the cell might make of this apparently-wasteful turnover. As a nuclear ribosome might proof-read nascent RNA for PTCs [3]–[4], turnover could reflect the destruction of peptides produced as a by-product. A test *Cd2* gene ± PTC (**Fig. S4**) was expressed using a multi-copy system; on transfection, the vector encoding *Cd2* replicates to generate several thousands of ‘mini-chromosomes’ that are co-transcribed by cellular polymerases in discrete foci [30]. *Cd2* expression is driven by a promoter normally switched off by a modified ‘Tet’ repressor (introduced by co-transfection with a second plasmid), but it can be switched on by doxycycline; then, levels of nascent *Cd2* RNA are monitored by fluorescence *in situ* hybridization (FISH) using probes targeting intronic regions of the RNA. As expected, the (control) PTC¯ vector expresses high levels of these intronic regions in many nucleoplasmic foci (**Fig. 4A,B**). The PTC^+^ vector yields less nuclear signal (**Fig. 4C** **i**); presumably, because the RNA has been destroyed by NMD. Significantly, cycloheximide increases signal above that seen in the control (**Fig. 4C** **i**) – consistent with it preventing ribosomes from detecting nascent PTC^+^ transcripts (so they are no longer destroyed by NMD). This places active ribosomes close to pre-mRNA which is found only in nuclei. [Ribosomes, NMD components, and proteasomes are all found in the nucleoplasm (often at transcription sites) at concentrations that roughly equal those found in the cytoplasm [31]–[32]].

The experiment described above involved an artificially-introduced PTC; but how might PTCs arise normally? One possibility is that they are introduced accidentally by faulty splicing; if a splice-site is missed, the intron remains in the message and any stop codons in it will be detected as PTCs [33]. Then, inhibiting splicing with spliceostatin A (SSA; [34]) should generate unspliced transcripts with intronic PTCs, and these should be degraded by NMD to reduce the FISH signal. Consistent with this, spliceostatin reduces the signal; moreover, anisomycin reverses this reduction (**Fig. 4C** **ii**). In other words, inhibiting a translating ribosome prevents detection of PTCs, and subsequent degradation of intron-containing RNA [21]. These results are consistent with a translating ribosome scanning nascent transcript for PTCs, and – once found – faulty transcripts and truncated peptides produced as by-products being degraded. As the peptide turnover is significant, it follows that many nascent RNAs must encode PTCs.

## Discussion

Initially, our aim was to re-examine whether any translation occurs in the nucleus, and to do so we applied three approaches utilizing different analogues and detection methods. In one, cells (transformed HeLa or diploid HUVECs) are pulse-labeled with Aha (a Met analogue), fixed, and a fluor ‘clicked’ on to the (nascent) Aha-labeled peptides [17]. In the second, cells are pre-treated with cycloheximide to ‘freeze’ ribosomes, pulse-labeled with puromycin (a structural mimic of aminoacyl-tRNA), fixed, and the now-puromycylated peptides immuno-localized using an anti-puromycin antibody [25]. In the third, we used a classical approach: cells are incubated in ‘heavy’ amino acids, and incorporated label analyzed by mass spectrometry (in our case, the spectrometer is incorporated into a microscope). To ensure most signal marks nascent peptides, we use pulses as short as 5 s (when ∼25 residues are incorporated into a typical protein that will contain ∼400 amino acids when complete). All three different methods give signal in both nucleus and cytoplasm, with the incorporation being sensitive to translation inhibitors (**Figs 1** **–** **3**). Surprisingly, the total amount of nuclear signal is only slightly less than the total amount seen in the cytoplasm. Extraordinarily, most signal in both compartments turns over in less than a minute (**Figs 1B**, **3B**) – and not over many hours as we expected [11]–[12].

We were concerned that the signal seen stems either from an adventitious binding of our labels to some unknown site within cells, and/or the incorporation of our labels by some unknown enzyme activity – and not from peptide bond formation by the ribosome. Results from the experiment described in **Figure 3C** make both possibilities unlikely. Thus, a peptide bond results from the covalent linkage of a (carboxyl) carbon in one amino acid with the (amino) nitrogen in another. Therefore, we incubated cells in both (heavy) [^13^C]amino acids and (heavy) [^15^N]amino acids; then, protein synthesis will covalently link some ^13^C atoms with ^15^N atoms in peptide bonds. Previous work has shown that heavy-heavy ions detected by mass spectrometry are generated with a higher probability when ^13^C and ^15^N atoms are directly bonded to each other in the same molecule [29]. After 2-min pulses, we find heavy-heavy ions in both nucleus and cytoplasm (**Fig. 3C**), consistent with the incorporation of these heavy labels into peptide bonds. As the relative levels of the heavy-heavy ions, as well as the effects of ribosomal inhibitors and chases on those levels, are essentially the same as those seen with all the other labels and the shorter pulses (i.e., the heavy Met/Lys/Arg, as well as Aha and puromycin; **Figs 1** **–** **3**), we think it fair to generalize that all our labels are incorporated by the ribosome into peptides in the expected manner.

Various additional arguments support this generalization. For example, could the incorporation of we see result from some non-ribosomal activity (e.g., through the action of a tRNA amino-acyl synthetase, or through α-amidation, trans-glutamination, or enzymes like an Arg-tRNA transferase or bacterial L/F transferase)? Probably not, as none of these non-ribosomal activities are sensitive to translational inhibitors [35]. Could our analogues be incorporated into (or attach to) molecules like DNA and RNA (e.g., through amino-acylation of a tRNA)? If so, the signal should be sensitive to DNase and RNase, and translation and proteasomal inhibitors should have no effect – but none of these apply (**Fig. 1C** **, Fig. S1**). Could the different analogues bind to other unknown molecules? Again probably not, as binding would have to be sensitive to translation inhibitors, the resulting complex would have to be degraded by the proteasome, and the concentration of that molecule would have to change in extraordinary ways during pulse-chases (**Figs 1B** and **3B**).

But if ribosomes are responsible, why have the synthesis and turnover not been detected previously? Consider an analogous situation. About 95% newly-made nuclear RNA is degraded almost as soon as it is made. This turnover was only discovered in the 1950s once labeling periods shorter than the half-life of the newly-made RNA were introduced, and no credible reason for why it might occur could be proposed at the time [15]. [Only now do we now know it results from the combined destruction of prematurely-terminating (abortive) transcripts and intronic plus non-coding RNA, and only now have ‘RNA-seq’ techniques sensitive enough to allow accurate measurement of these unstable RNAs been developed.] Although such synthesis and destruction of RNA appears wasteful, it is nevertheless an integral part of cell metabolism. By analogy, we suggest the extraordinary turnover of most newly-made peptides went undetected because pulses longer than the half-life were used. [Previous work puts a lower limit of ∼30% on the fraction of newly-made peptide that is destroyed within minutes [10], [36].] We also suggest that if many ribosomes abort soon after initiation, it is likely that the resulting short peptides will be missed using conventional approaches. Some possible reasons for this include: (i) translation was traditionally monitored using amino acids tagged with radiolabels, followed by acid precipitation of any now-tagged proteins – but short peptides are not precipitated using standard conditions [37], (ii) peptides containing fewer than ∼10 amino-acid residues go undetected in conventional proteomic screens, and (iii) current biochemical approaches are insufficiently sensitive to permit detection of analogues incorporated during the short pulses used here (see **Materials and methods** for one approach we tried).

These results beg the question: does the cell utilize this apparently-wasteful turnover? In the case of the nuclear turnover, it seems it does. Thus, early results [3]–[4], and those in **Figure 4**, are consistent with a nuclear ribosome proof-reading nascent RNA for PTCs, and – if found – degradation of the RNA by NMD. Then, a truncated peptide is an inevitable by-product, and – as this may be toxic – we suggest it is quickly degraded.

**Figure 5:**
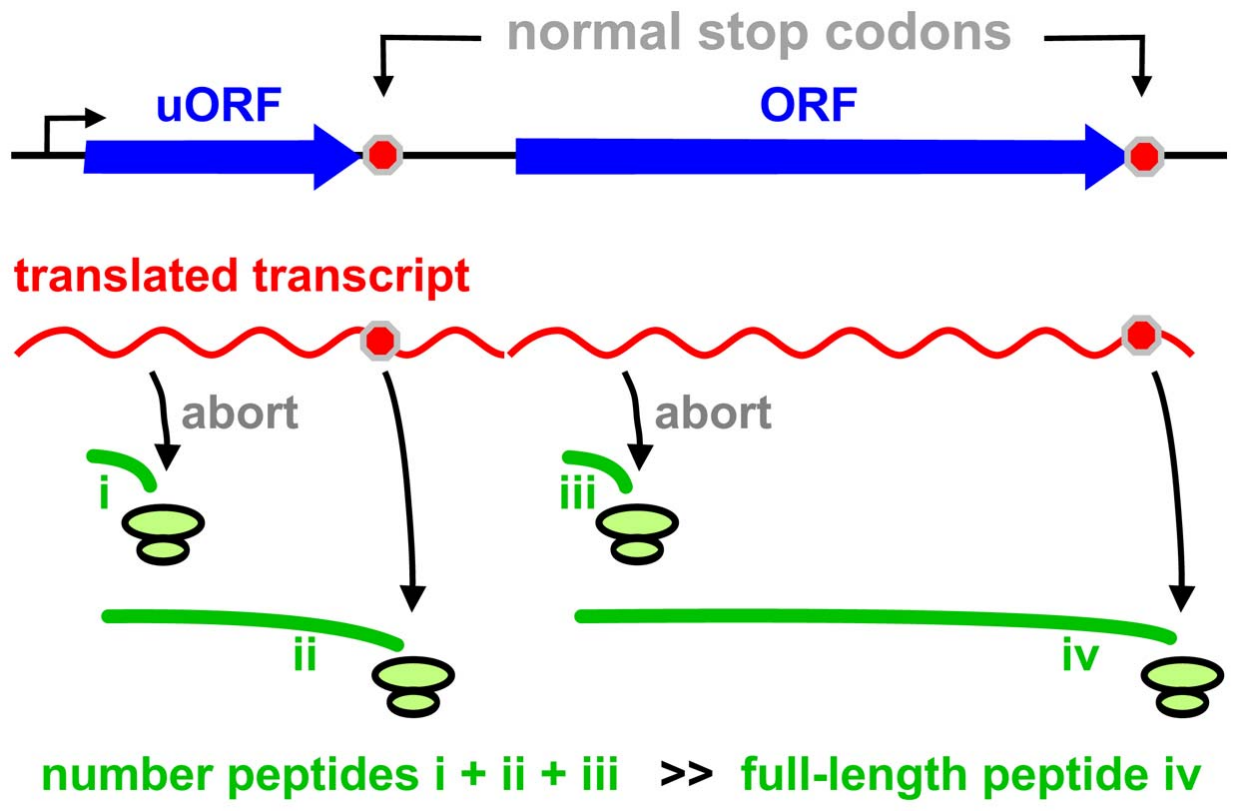
A model illustrating how ‘dark-matter’ peptides (green lines i-iii) and the ‘mature’ proteome (iv) arise. Most initiated ribosomes terminate prematurely (giving i and iii), and some translate to the end of an uORF (giving ii); the resulting peptides are rapidly degraded (half-life <1 min), to give rise to the astonishing turnover seen using short pulses. A minority of ribosomes translate the whole ORF (giving iv); such peptides are the ones detected conventionally using long pulses (they are generally stable and constitute the ‘mature’ proteome). During long pulses, most peptides i-iii are degraded and so are not detected.

But how might the cell utilize the cytoplasmic turnover? We can only speculate. First, nuclear proof-reading may be so error-prone the system has a second go with a cytoplasmic ribosome at weeding out unwanted PTC^+^ transcripts. Second, more proteins than hitherto expected may terminate prematurely and/or misfold, and these are probably degraded quickly [10], [13]–[14], [38]. Third, and in the special case of antigen-presenting cells, some newly-made peptides may be used to fight infection [39]. Finally, the apparently-wasteful turnover could play an important role in regulating translation. Here, RNA synthesis again provides a precedent. Thus, for every ∼100 RNA polymerases that initiate at a promoter, ∼99 will abort to release transcripts of 2–15 nucleotides [40]. These transcripts are not detected using high-throughput sequencing, as they are too short to be mapped. Subsequently, many of the polymerases that manage to ‘escape’ without aborting will now terminate within 20–500 nucleotides to give the sense and anti-sense transcripts copied from in/around promoters that can be detected by high-throughput sequencing [41]. As a result, the production of each completed transcript is regulated by the synthesis of hundreds of shorter sense and anti-sense products. Although this synthesis and destruction may appear energetically wasteful to us, the associated costs must be marginal – simply because the turnover is an integral part of metabolism, and because the system then goes on to discard nine-tenths of the resulting full-length transcript when introns are removed to give the mature mRNA. We suggest the same general principles apply to translation. Here, we also note that ∼50% human mRNAs encode upstream open-reading frames (uORFs; [42]) that ribosome profiling indicates are translated [43]–[44] – presumably into peptides containing only a handful of residues that we also assume must be quickly degraded [13]–[14], [44]–[45]. Moreover, many of these uORFs are both conserved and play a role in regulating translation of their associated ORFs [43]–[45]. If translation is like transcription, then – during our short pulses – many ribosomes will terminate soon after initiation (both at the end of uORFs, and close to the beginning of uORFs and ORFs), most peptides generated in this way will be degraded quickly, and only a minority of ribosomes will go on to generate the peptides that contribute to the ‘mature’ proteome (**Fig. 5**). [Many of these ribosomes that terminate quickly may only be weakly associated with the mRNA, and so be lost during the cell lysis and treatment with the RNase used during ribosome profiling. This could explain why profiling uncovers only a small peak of bound ribosomes near initiation codons, and why translation inhibitors increase the height of this peak (i,e., by ‘freezing’ an unstable fraction on the message) [43], [46].] Cycles of initiation and abortion will lead to preferential incorporation of Aha or [^15^N]Met, and to the astonishing turnover we see (because these are end labels, as well as internal labels); in contrast, incorporation of [^15^N]Lys and [^15^N]Arg will label more of the ‘mature’ proteome (because they are incorporated by the ribosome only internally). And conventional pulses lasting more than a few minutes (with end or internal labels) will completely miss the soon-to-terminate fraction.

Then, protein synthesis is like RNA synthesis: pulse-labels of different times highlight different metabolic pools, most newly-made material is degraded as soon as it is made, and the apparently-wasteful turnover is a by-product of an inefficient process. Whilst most of this turnover probably reflects ‘noise’, it seems likely that some will be exploited by the system to regulate production. Then, a hitherto unrecognized – and so ‘dark-matter’ – world of short peptide will coexist with its RNA counterpart [47].

## Materials and Methods

### Cell culture and fixation

General conditions are described, with exceptions indicated in the legends to individual Figures. HeLa and Cos 7 cells were cultured in DMEM (PAA) supplemented with 1% penicillin/streptomycin (PAA) and FBS (Bioline; 5% for HeLa, 10% for Cos 7); HUVECs (human umbilical vein endothelial cells; Lonza; note that the supplier were responsible for obtaining the necessary ethical provision) were cultured in EGM-2 (Lonza). Prior to imaging, cells were cultured on clean glass coverslips (22×22 mm; no. 1.5) etched with 1% hydrofluoric acid (Merck). 2 mM Aha (Invitrogen, or synthesized as described in Materials and Methods S1), 100 µg/ml anisomycin (Sigma), 100 µg/ml MG132 (Enzo), 91 µM puromycin (Sigma), 100 µM DRB (Sigma), 10 µM doxycycline (Calbiochem), 100 µg/ml cycloheximide (Sigma), and 100 nM spliceostatin A (a gift of M Yoshida) were added to live cells at the times indicated. Prior to pulsing with Aha, HeLa cells were grown in the absence of Met (i.e., in DMEM¯, which is DMEM without Met, cysteine, and L-glutamine; Sigma) for 15–30 min at 37°C (to deplete endogenous pools of Met). Prior to pulsing with puromycin, cells were grown in the presence of cycloheximide (in DMEM supplemented with 5% FBS and 1% penicillin/streptomycin for HeLa, and EGM-2 for HUVECs) for 15 min at 37°C (to slow translating ribosomes). For pulses of 5–120 s in Aha or puromycin (and for pulse-chase experiments), cells on a coverslip were dipped in medium (DMEM¯ for HeLa and EGM-2 for HUVECs) containing the analogue (and then into medium lacking the analogue during a chase) in a 37°C room for the required time before plunging the coverslip immediately into ice-cold fixative. When a pulse was followed by chase, DMEM¯ +2 mM Aha was replaced by DMEM (which contains ∼200 µM Met) supplemented with 5% FBS and 1% penicillin/streptomycin, or EGM-2 in the case of HUVECs. Cells were fixed with 4% paraformaldehyde (Electron Microscopy Sciences) in 250 mM HEPES (pH 7.6; PAA) for 20 min, usually at 20°C. For transfection, Cos 7 were grown in DMEM supplemented with 10% FBS (tetracycline free; Clontech) to 80–90% occupancy in 6-well plates and transfected using 8 µl FuGENE HD (Roche/Promega) with 2 µg DNA that included 0.2 µg of Vector 1−3+0.2 µg pTetOn Advanced (Clontech) +1.6 µg sheared salmon sperm DNA (Invitrogen). Vectors are described in Materials and Methods S1. After 12 h, cells were trypsinized, washed, and replated on coverslips in 6-well plates (to reduce background caused by input DNA). Cells were then grown for 12 h, induced with doxycycline in the presence or absence of inhibitors, and fixed.

### Fluorescent detection of Aha by ‘click’ chemistry

After fixation, cells were washed in PBS (PAA; 5 min; 20°C), permeabilized with 0.5% Triton X-100 (Sigma), washed in 3% BSA in PBS (5 min; 20°C), an alkyne-conjugated fluor (i.e., 100 nM Alexa555; Invitrogen) attached (using the copper-catalyzed azide-alkyne cycloaddition reaction, CuAAC) to incorporated Aha using the ‘Click-iT Cell Reaction Buffer kit’ (Invitrogen) as instructed by the supplier (30 min; 20°C; vigorous shaking), washed in 3% BSA in PBS (5 min; 20°C), and rinsed in PBS. After counterstaining nuclei with DAPI (Sigma) and mounting in Vectashield (Vector Laboratories), images were acquired images using a wide-field or confocal microscope.

### Immuno-labeling

Fixed cells were washed with PBS (5 min; 20°C), permeabilized with 0.5% Triton X-100 and 0.5% saponin (Sigma), washed with 0.05% Tween 20 (Sigma) in PBS (10 min; 20°C), and blocked with 3% BSA and 0.2% cold water fish skin gelatin (Sigma). Puromycylated peptides were indirectly immuno-labeled by incubation (1 h; 20°C) with a mouse monoclonal antibody, 12D10 (1/100 dilution), cells washed 4x with 0.05% Tween 20 in PBS (1 min each; 20°C), incubated (30 min; 20°C) with a Cy3-conjugated donkey anti-mouse (Jackson ImmunoResearch, 715-165-150; 1/200 dilution), and washed 3x with 0.05% Tween 20 in PBS (10 min each; 20°C) followed by a wash with PBS (10 min; 20°C). After staining with DAPI and mounting in Vectashield, images were acquired with a confocal or wide-field microscope.

### Fluorescence microscopy

Microscopes used were: (a) Wide-field fluorescence microscope (Zeiss; upright – Axioplan 2e microscope), equipped with a 175 W Xenon arc lamp (Perkin Elmer); images were acquired using a 63x Zeiss Plan-APOCHROMAT oil-immersion objective (numerical aperture 1.4), optical filters (Chroma), and a CoolSNAP*_HQ_* camera (Photometrics) running under MetaMorph software (Molecular Devices). (b) Confocal microscope (Olympus, FV1000, IX81); images were acquired using a 100x Olympus UPlanSApo oil-immersion objective (numerical aperture 1.4), optical filters (Olympus), a confocal aperture of 175 µm and scanning at 10 µs/pixel, 405 and 559 nm ‘diode-pumped solid-state’ or argon (488 nm) lasers, and FLUOVIEW v2.1b software (Olympus). Images were analyzed using ImageJ [48].

The fluorescence intensity over nucleus and cytoplasm (images obtained using a wide-field microscope) was determined as follows. (i) The fluorescent intensity over the whole cell and nucleus (area defined by DAPI staining) was measured, and that over the cytoplasm calculated by subtraction. (ii) Background in each compartment was subtracted; backgrounds determined separately for nucleus and cytoplasm (i.e., whole cell minus nucleus) over non-pulsed, 0-s pulsed, or untransfected cells as appropriate. (iii) Values from each experiment were exported to Excel, and mean and standard deviation (SD) calculated. *P* values (two-tailed) from unpaired Student’s *t*-test were calculated using GraphPad (http://www.graphpad.com/quickcalcs/). For **Figure 4**, the mean intensity within a randomly-selected 30×30 pixel area over the nucleoplasm was measured.

### Detecting Aha-tagged peptides biochemically

It is generally accepted that fluorescence microscopy can allow detection of fewer tagged molecules than conventional biochemical approaches. Nevertheless, we attempted to detect nascent Aha-tagged after running them on gels, but failed to do so successfully. For example, one robust approach we tried involved combining two analogues: after 60-s growth in Aha (with puromycin added during the last 5 s), clicking on a cleavable biotin (i.e., compound 14b from [49]), selection on Neutravidin beads, release of biotinylated peptides (using 0.5 M dithiothreiotol, and ‘Western’ blotting using the anti-puromycin antibody, we failed to distinguish between signal and background.

### NanoSIMS

HeLa cells were grown on coverslips. For labeling with heavy Lys and Arg (**Fig. 3A**, **Fig. 3B** **ii**), cells were grown in custom-made DMEM without Lys + Arg (Thermo Scientific) for 30 min, regrown in DMEM (without Lys + Arg) supplemented with 8 mM [^15^N^13^C]Lys +4 mM [^15^N^13^C]Arg (Cambridge Isotopes Laboratories; ^15^N 99% enriched) for 10 or 120 s at 37°C, immediately plunged into ice-cold fixative, and incubated in fixative for 20 min at 4°C (see below). [The ^13^C tag is not used here.] For labeling with heavy Met (**Fig. 3B** **i**), cells were grown in DMEM without Met and cystine (Sigma) for 30 min, regrown in the same medium supplemented with 2 mM [^15^N]Met (Cambridge Isotopes Ltd; ^15^N 97–99%) for 2 min, and fixed (see below). For labeling with [^13^C]amino acids plus [^15^N]amino acids (**Fig. 3C**), cells were starved for 15 min in custom-made DMEM lacking any amino acids (Thermo Scientific), regrown in the same medium supplemented with [^13^C]algal amino acid mixture (Isotec; uniformly ^13^C-labeled, 98 atom % ^13^C) plus [^15^N]cell-free amino acid mixture (Cambridge Isotope Labs Inc; uniformly ^15^N-labeled, 96.98%), and fixed (see below). Here, we assume each of the 20 amino acids has a molecular weight of 136, and that each kind summed to give a final concentration of 1 mM during the pulse. In some cases, (i) 100 µg/ml anisomycin or 100 µg/ml cycloheximide was present during both the starvation and the pulse, and (ii) the pulse was followed by chases in DMEM plus 5% fetal bovine serum at 37°C or 4°C.

After the pulse, or pulse-chase, sections of cells were prepared using a procedure commonly used for electron microscopy. Cells were fixed in 4% paraformaldehyde (Electron Microscopy Sciences) and 1% glutaraldehyde (Agar) in PBS (PAA). Samples were further fixed (1 h; room temperature) with 2.5% glutaraldehyde in 0.1 M sodium cacodylate buffer (pH 7), washed in the buffer 3x (10 min each), incubated (1 h) in 1% osmium tetroxide in the same buffer, and washed with water for 20 min. Samples were dehydrated using a graded ethanol series (50%, 70%, 90%, 95% and 100%), and embedded in Agar100 epoxy resin; after removing the coverslip, semi-thin sections (0.5 µm) were cut using an ultramicrotome (Leica UC7) with a diamond knife (Diatome), mounted on platinium-coated coverslips, and coated with 5-nm platinum.

SIMS images were acquired on a CAMECA (Gennevilliers) NanoSIMS 50 to measure the ^15^N and ^13^C signals in the nucleus and cytoplasm. The NanoSIMS uses a 16 keV Cs^+^ ion beam to bombard the sample surface, and was tuned to detect ^12^C^14^N^−^, ^12^C^15^N^−^, and ^13^C^15^N^−^ ions using a Mauttach-Herzog mass analyser with electrostatic sector and asymmetric magnet configuration. The instrument was tuned to avoid mass interference from ^12^C^14^N^−^ and ^12^C^15^N^−^ ions as appropriate. Images were analysed using the Image J plugin OpenMIMS (MIMS, Harvard University, www.nrims.harvard.edu). Regions of interest (ROIs) were selected in the nucleus and cytoplasm that avoided nucleoli and obvious cytoplasmic vesicles or oil droplets, and ratios calculated as indicated (i.e., ^12^C^15^N^−^/[^12^C^15^N^−^+^12^C^14^N^−^], and ^13^C^15^N^−^/^12^C^14^N^−^). The control sample in **Figure 3A** **iii** *row 1* had a ^12^C^15^N^−^/[^12^C^15^N^−^+^12^C^14^N^−^] ratio of 0.003838, which compares well with the expected value of 0.003667 (i.e., the ratio of ^15^N/^14^N in the biosphere); therefore other ratios were normalized relative to this expected value.

### RNA fluorescent *in situ* hybridization (RNA FISH)

A set of forty-eight 20-mer probes (designed using Probe Designer; http://singlemoleculefish.com/) were synthesized (Biosearch Technologies, USA) targeting the intronic region (∼1.6 kbp) of rat *Cd2* DNA present in Vectors 1–3 (**Fig. S4**; see Materials and Methods S1 for vector construction, and **Table S1** for the sequences of primers used during construction). At the 3′-end of each 20-mer, an mdC(TEG-amino) modification was added. The amino group was subsequently labeled [50] using the ARES Alexa Fluor 488 DNA labeling kit (Invitrogen) according to the manufacturers' instructions. Labeling efficiency was found to be ∼5 fluors per 100 nucleotides. Fixed cells were processed for RNA FISH (probe sequences are listed in **Table S2**), and imaged as described [50] with the following exceptions: cells were immediately permeabilized as above for immuno-fluorescence, and the hybridization buffer contained 10% formamide. When RNA FISH was performed on EGFP-expressing cells, Alexa488 fluor was further detected by immuno-fluorescence as follows. After performing RNA FISH as described above, coverslips were washed 3x in 2xSSC (10 min each; 37°C), washed with 0.05% Tween 20 in PBS (3 min; 20°C), and blocked as above. The *Cd2* intronic RNA was detected by incubating the cells with primary antibody against Alexa 488 Fluor (rabbit polyclonal; Invitrogen, A11094; 1 µg/ml; 1 h; 20°C), washed 4x with 0.05% Tween 20 in PBS (1 min each; 20°C), then labeled further by incubating these cells with Cy3-conjugated donkey anti-rabbit (Jackson ImmunoResearch, 711-165-152; 1/2000 dilution; 30 min; 20°C), washed 4x with 0.05% Tween 20 in PBS (5 min each; 20°C) followed by a rinse with PBS at (5 min; 20°C). After DAPI/Vectashield counter-staining and mounting, images were collected using a wide-field microscope.

## Supporting Information

Figure S1 **Aha incorporation; some controls.**
(TIF)

Figure S2 **Comparison of images obtained using wide-field and confocal microscopes.**
(TIF)

Figure S3 **Puromycin incorporation: some controls.**
(TIF)

Figure S4 **CD2-EGFP expression constructs.**
(TIF)

Table S1 **Oligonucleotide DNA primers.**
(PDF)

Table S2 **RNA FISH probes against rat** ***Cd2*** **intron-2^a^.**
(DOCX)

Materials and Methods S1 (DOCX)
