# Supplementary figures and images for: Most Human Proteins Made in Both Nucleus and Cytoplasm Turn Over within Minutes
Source: PLoS One. 2014 Jun 9;9(6):e99346. doi: 10.1371/journal.pone.0099346 (PMC4050049; doi:10.1371/journal.pone.0099346)

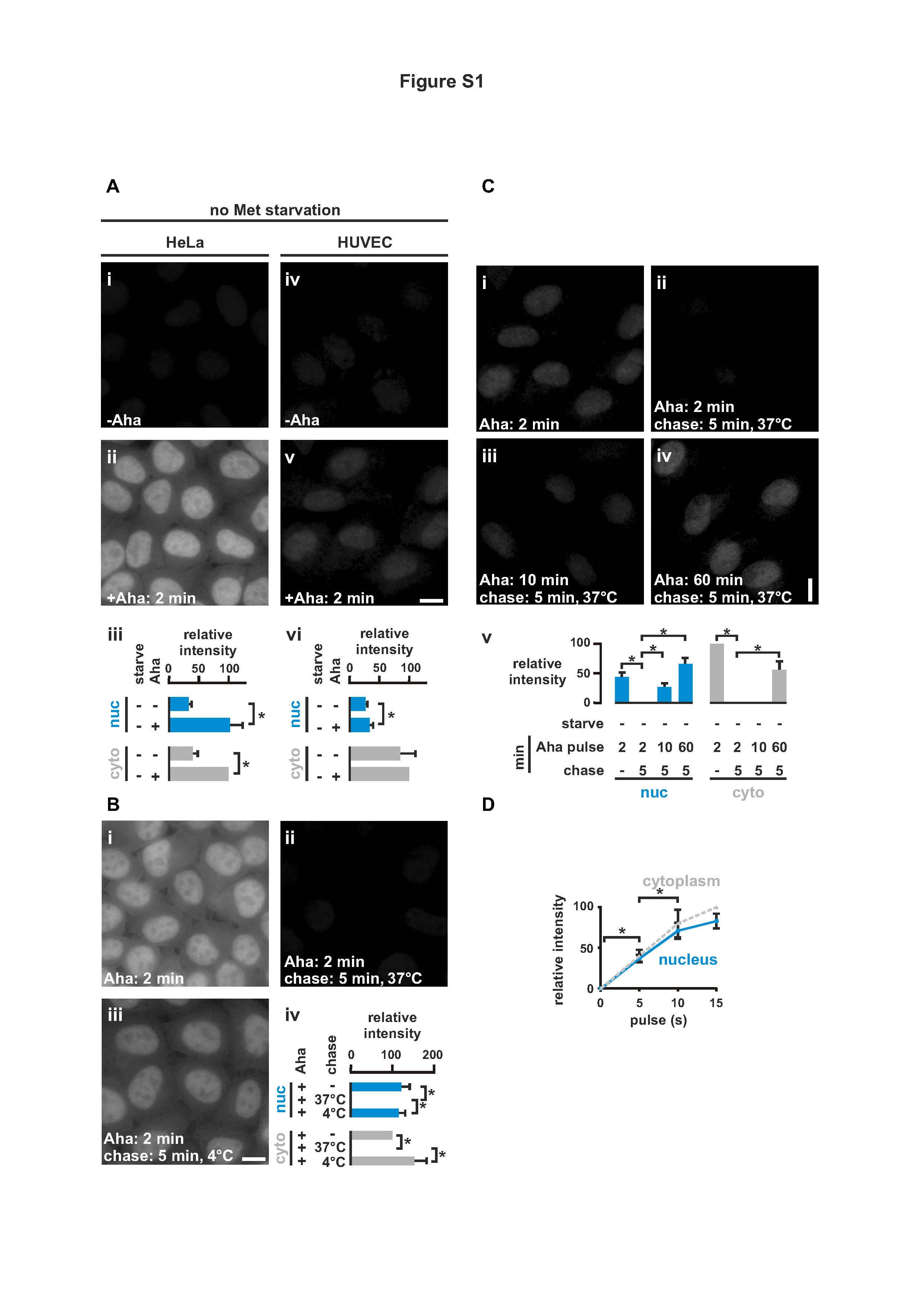

Supplement: Figure S1 — Aha incorporation; some controls. (TIF) [file pone.0099346.s001.tif]

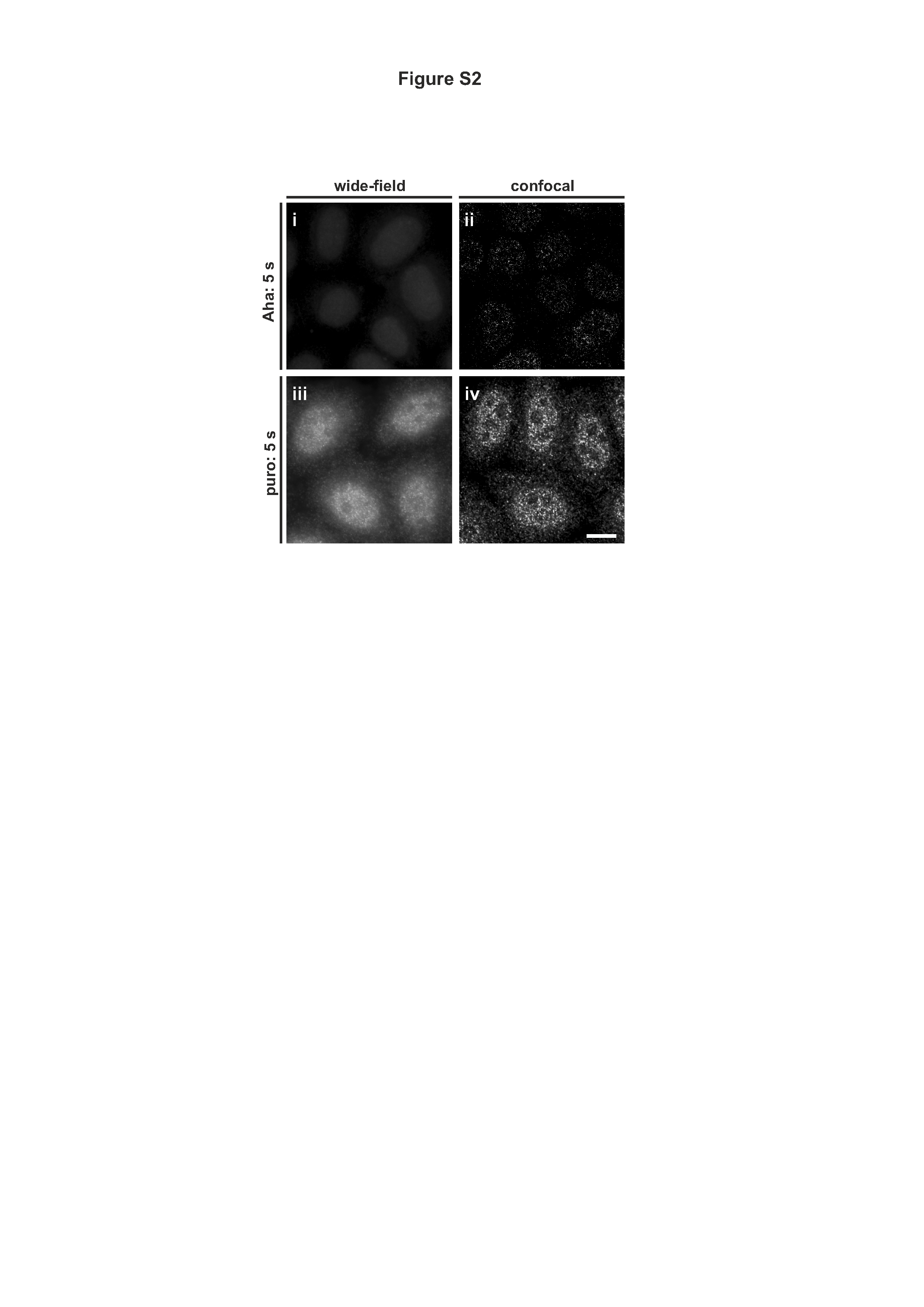

Supplement: Figure S2 — Comparison of images obtained using wide-field and confocal microscopes. (TIF) [file pone.0099346.s002.tif]

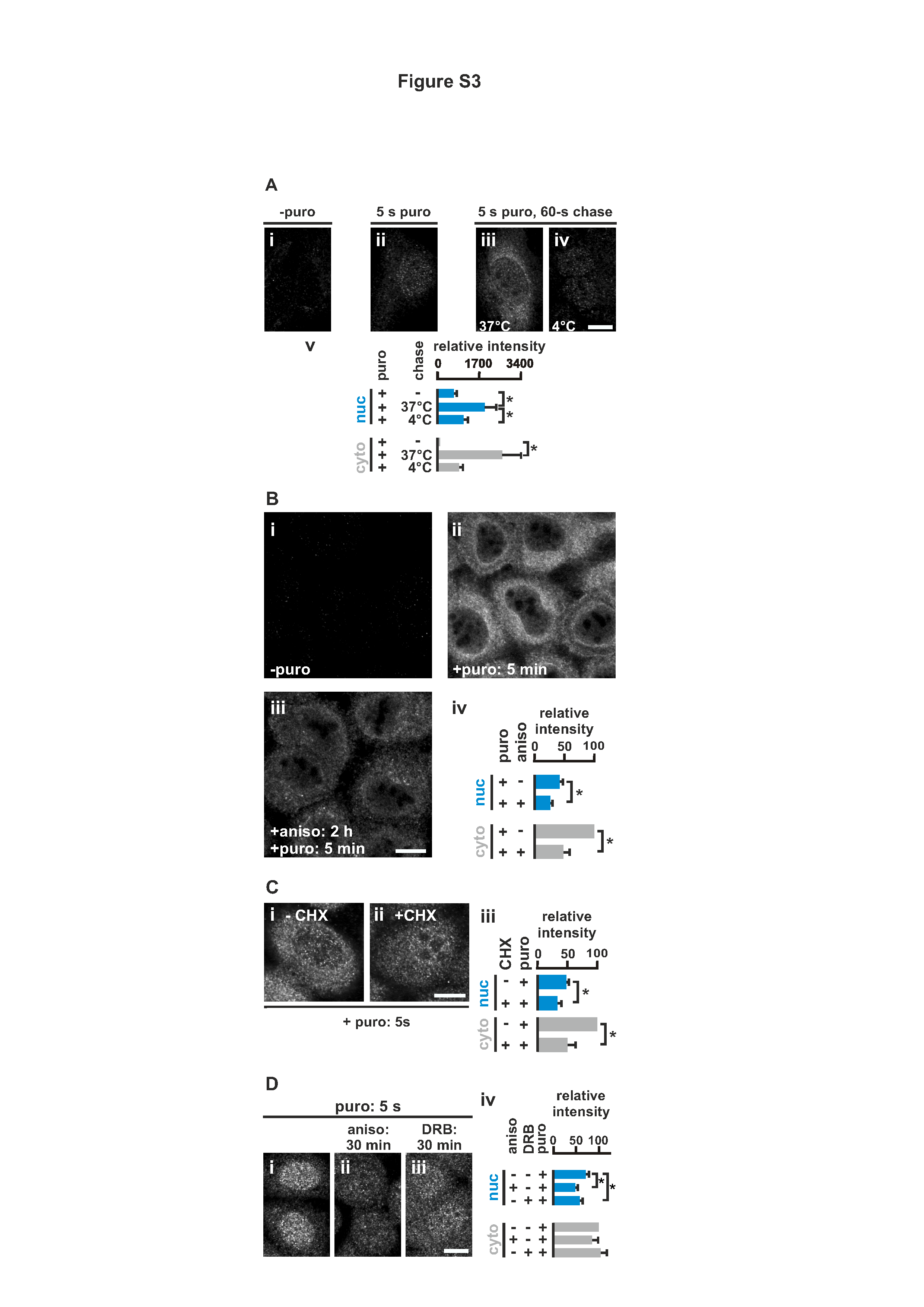

Supplement: Figure S3 — Puromycin incorporation: some controls. (TIF) [file pone.0099346.s003.tif]

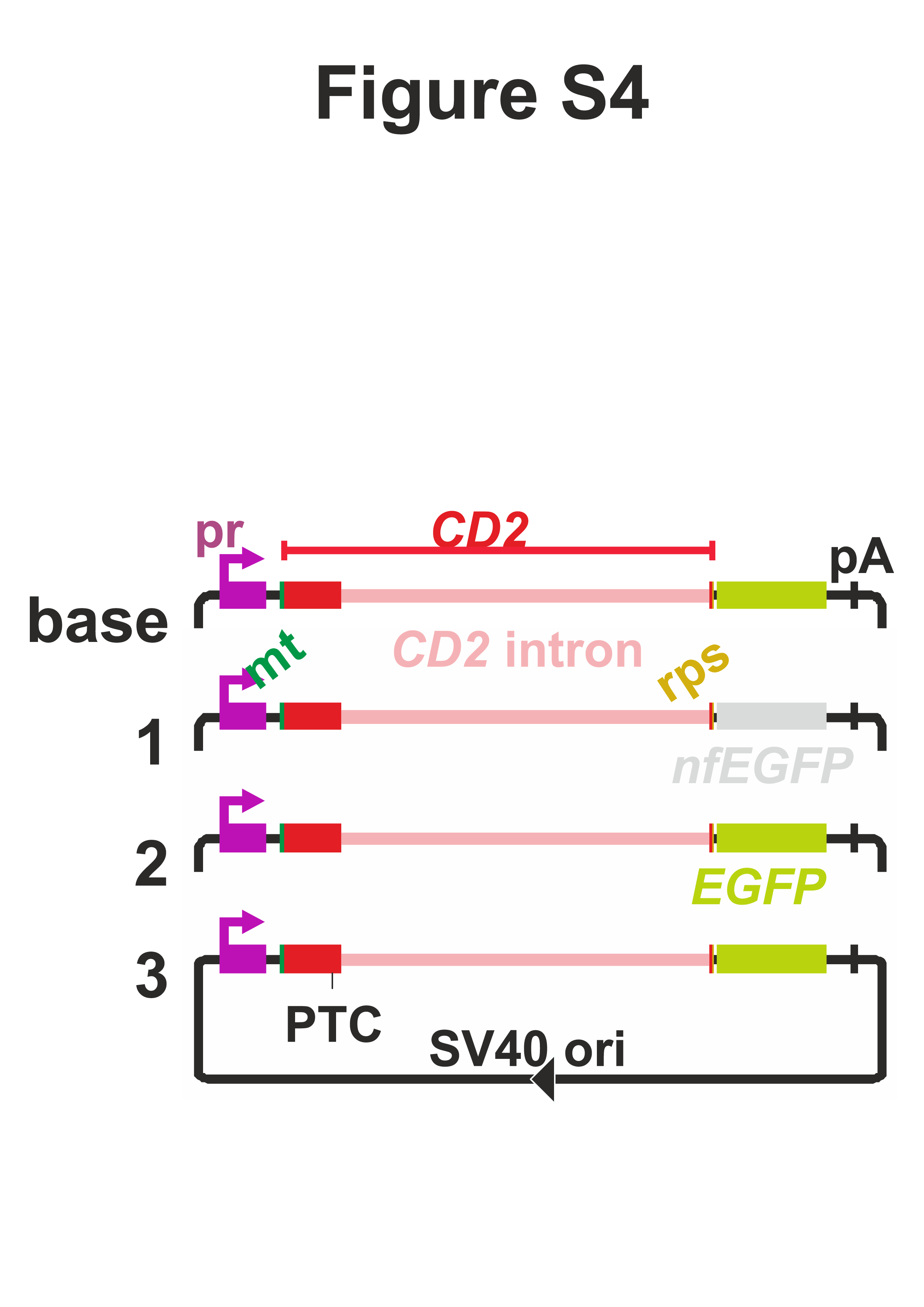

Supplement: Figure S4 — CD2-EGFP expression constructs. (TIF) [file pone.0099346.s004.tif]
