# Supplementary material for: Most Human Proteins Made in Both Nucleus and Cytoplasm Turn Over within Minutes
Source: PLoS One. 2014 Jun 9;9(6):e99346. doi: 10.1371/journal.pone.0099346 (PMC4050049; doi:10.1371/journal.pone.0099346)
Supplement: Table S1 — Oligonucleotide DNA primers. (PDF) [file pone.0099346.s005.pdf]

Table S1. Oligonucleotide DNA primers.

#	Name	Sequence (5′-3′)
1	Oligo pair 1	Fwd: GCAGA(CTGCAG)[b]AGAGACAGTGG<br>Rev: CAGCATCTCCAGCCTTCTTC
2	Oligo pair 2	Fwd: ATCCACAGAAAGGTGCTCGT<br>Rev: TA(GGATCC)[c]AT(TGTTCGTCGTCG)[d]CTTTGAGACCATCTCTAAAAG
3	Oligo pair 3	Fwd: AC(TCTAGA)[e]AAGACAGGCAGTCTCTG<br>Rev: TA(GGATCC)[c]AT(TGTTCGTCGTCG)[d]CTTTGAGACCATCTCTAAAAG
4	Oligo pair 4[a]	Fwd: CACCCTCGTGACCACCTGCTTCAGCCGCTAC<br>Rev: GTAGCGGCTGAAGCAGGTGGTCACGAGGGTG
5	Oligo pair 5[a]	Fwd: GGACGGGTGCTATG(TACCCTTACGACGTTCCTGATTACGCT)[f]TAGCAAGTTGGG<br>Rev: CCCCAACTTGCTA(AGCGTAATCAGGAACGTCGTAAGGGTA)[f]CATAGCACCCGTCC
6	Oligo pair 6[a]	Fwd: GAGATGACAGTGGCACC(TAA)[g]AATGTAACGGTATACAGCAC<br>Rev: GTGCTGTATACCGTTACATT(TTA)[g]GGTGCCACTGTCATCTC
7	Oligo 7	GCCATCATCCAACAGAGCAG
8	Oligo 8	CGTCGCCGTCCAGCTCGACCAG
9	Oligo 9	TGCCCTTCAGCTCGATGCGG

[a]: complimentary oligonucleotide primers for site-directed mutagenesis, HPLC purified

[b]: *PstI* recognition sequence

[c]: *BamHI* recognition sequence

[d]: ribosome pause sequence (rps)

[e]: *XbaI* recognition sequence

[f]: HA-epitope encoding sequence

[g]: premature termination codon (PTC)
