# Supplementary material for: Most Human Proteins Made in Both Nucleus and Cytoplasm Turn Over within Minutes
Source: PLoS One. 2014 Jun 9;9(6):e99346. doi: 10.1371/journal.pone.0099346 (PMC4050049; doi:10.1371/journal.pone.0099346)
Supplement: Table S2 — RNA FISH probes against rat Cd2 intron-2a. (DOCX) [file pone.0099346.s006.docx]

**Table S2.** RNA FISH probes against rat *Cd2* intron-2^a^.

| **#** | **Sequence (5ʹ-3ʹ)** | **#** | **Sequence (5ʹ-3ʹ)** |
| --- | --- | --- | --- |
| 1 | GCACCCGTCCTAAAATGAAA | 25 | CTCCAAGGTTCTGAGTTCAT |
| 2 | TGTTACAGAAACGCTACTCC | 26 | TTACCAGAGTAAAGGCACCA |
| 3 | AACAAGAAACGACAGACAGG | 27 | AATGACTACATGAGAGGCCT |
| 4 | ACTGGAGTCTTCATTGTGAG | 28 | AGTGTAGGTGAAGTCAGTCA |
| 5 | AAGGAAAGGCAGACAGACTT | 29 | CACAAAGGATTCCCAAGTAC |
| 6 | AACCTGGGAGTCTTTACACA | 30 | ATGTCAGGCCAGAAGAAGAA |
| 7 | GCCGACCTGTTTTCTATCTT | 31 | CACCCGGGAATACAATTGTT |
| 8 | CAAGGATGTCCACCTTTATC | 32 | TACACACACACACACACACA |
| 9 | TGTCTACAACTTCATCAGCC | 33 | ATGTTGTAGATGCATGCGTG |
| 10 | TTAGCTCTCCAGACAAGAGA | 34 | TTTGCTGTTCTTCCAGAGGA |
| 11 | ATCTTGCCCTCTAACTCCTT | 35 | GTTGGCTGAACAGTTAAGAG |
| 12 | AGAGACTGCCTGTCTTTCTA | 36 | TAAGTACGACAAGTCAGAGC |
| 13 | ACTTGCCATCATCCAACAGA | 37 | AATGCCTAGCTTTGGGGTAA |
| 14 | GTCCTGAGTTTCTGTGTATC | 38 | CGTCCAAAGCTAATTGACCT |
| 15 | GCATGCAGAAATGCATTTCC | 39 | GTCTTCCCTTTTGCAGATGA |
| 16 | CATTTCTGCCTCCAACATAC | 40 | TCTAACAGCAAGCCTTCTGT |
| 17 | ATAGGCCTCCTTGGTGTAAT | 41 | TAAGCTGTGTCTCTACTGTG |
| 18 | TCAGATCACAGTGTCTTTCC | 42 | CAGGATGCGAGTAATATAGG |
| 19 | TTTTCCGATTTCCCCTCTCT | 43 | GACCTAAAGCATCTTGAGCT |
| 20 | TCAGTGGTCTCCATTCATCA | 44 | CCATCGAAGCTCTTTTGAAG |
| 21 | TTATTGAGATCAGGTCCAGG | 45 | GGACAAGAATCCTACCAACT |
| 22 | TCACACTGCAAATTCCACAC | 46 | AAACCCAGGTTTTCCTGCAT |
| 23 | ATTCCCTACCTGTCTCAAAC | 47 | TTTGCCAAGCTTCATGTGGT |
| 24 | GACTTGAAGGACCTCAACTT | 48 | TATTCTGACTCTCCCTCTAC |

^a^: probes against the +ve strand.
