# Supplementary material for: Most Human Proteins Made in Both Nucleus and Cytoplasm Turn Over within Minutes
Source: PLoS One. 2014 Jun 9;9(6):e99346. doi: 10.1371/journal.pone.0099346 (PMC4050049; doi:10.1371/journal.pone.0099346)
Supplement: Materials and Methods S1 — (DOCX) [file pone.0099346.s007.docx]

**SUPPLEMENTARY INFORMATION**

**MATERIALS AND METHODS**

**Synthesis of L-azidohomoalanine (Aha)**

Aha was synthesized in three steps: (i) *N*-tert-butoxycarbonyl-(S)-2,4-diaminobutanoic acid was synthesized (adapted from [51]) from *N*-tert-butoxycarbonyl-(*S*)-glutamine (Sigma). (ii) *N*-tert-butoxycarbonyl-(*S*)-azidohomoalanine was synthesized (adapted from [52]) from *N*-tert-butoxycarbonyl-(S)-2,4-diaminobutanoic acid. (iii) Aha was synthesized [53] from *N*-tert-butoxycarbonyl-(*S*)-azidohomoalanine. Properties of the products in steps 2 and 3 were compared with those made previously [54].

Melting points (m.p.) were recorded on a Leica Galen III hot stage microscope equipped with a Testo 720 thermocouple probe and are uncorrected. Proton nuclear magnetic resonance (^1^H NMR) spectra were recorded on a Bruker AVII500 (500 MHz) or on a Bruker DQX400 (400 MHz) spectrometer, as indicated. Carbon nuclear magnetic resonance (^13^C NMR) spectra were recorded on a Bruker AVII500 (125 MHz) or on a Bruker DQX400 (100 MHz) spectrometer, as indicated. NMR Spectra were fully assigned using COSY, HSQC, HMBC, and NOESY. All chemical shifts are quoted on the *δ* scale in ppm using residual solvent as the internal standard (^1^H NMR: CDCl_3_ = 7.26, CD_3_OD = 4.87; DMSO-*d*_6_ = 2.50 and ^13^C NMR: CDCl_3_ = 77.0; CD_3_OD = 49.0; DMSO-*d*_6_ = 39.5). Coupling constants (*J*) are reported in Hz with the following splitting abbreviations: s = singlet, d = doublet, t = triplet, q = quartet, quin = quintet, and a = apparent.

Infrared (IR) spectra were recorded on a Bruker Tensor 27 Fourier Transform spectrophotometer using thin films on NaCl plates for liquids and oils and KBr discs for solids and crystals. Absorption maxima (*υ*_max_) are reported in wavenumbers (cm^–1^). For compound characterization low resolution mass spectra (LRMS) were recorded on a Waters Micromass LCT Premier TOF spectrometer using electrospray ionization (ESI) and high resolution mass spectra (HRMS) were recorded on a Bruker MicroTOF ESI mass spectrometer. Nominal and exact *m/z* values are reported in Daltons. Optical rotations were measured on a Perkin–Elmer 241 polarimeter with a path length of 1.0 dm and are reported with implied units of 10^–1^ deg cm^2^ g^–1^. Concentrations (c) are given in g/100 ml. Thin layer chromatography (TLC) was carried out using Merck aluminium backed sheets coated with 60F254 silica gel. Visualization of the silica plates was achieved using a UV lamp (λ_max_ = 254 nm), and/or ammonium molybdate (5 % in 2 M H_2_SO_4_), and/or potassium permanganate (5 % KMnO_4_ in 1 M NaOH with 5 % potassium carbonate). Flash column chromatography was carried out using BDH 40–63 μm silica gel (VWR). Mobile phases are reported in relative composition (*e.g.* 1:2:4 H_2_O/*i*PrOH/EtOAc). Anhydrous solvents were purchased from Fluka or Acros. Triethylamine was stored over NaOH pellets. All other solvents were used as supplied (Analytical or HPLC grade), without prior purification. Distilled water was used for chemical reactions and Milli–QR purified water for protein manipulations. Reagents were purchased from Aldrich and used as supplied, unless otherwise indicated. ‘Petrol’ refers to the fraction of light petroleum ether boiling in the range 40–60 ºC. All reactions using anhydrous conditions were performed using flame-dried apparatus under an atmosphere of argon or nitrogen. Brine refers to a saturated solution of sodium chloride. Anhydrous magnesium sulfate (MgSO_4_) was used as drying agents after reaction workup, as indicated. DOWEX 50WX8 (H^+^ form) was conditioned as follows: 100 g of the commercial resin was placed in a 500 mL sintered filter funnel and allowed to swell with 200 mL of acetone for 5 minutes. The solvent was removed by suction and the resin was washed successively with 800 mL of acetone, 500 mL methanol, 500 mL 5 M HCl, and then 1 L of water or until the pH of filtrate was ~ 7, as indicated by pH paper. The resin was partially dried on the filter and then stored and used as needed.

The three steps were carried out as follows:

1. *N*-tert-butoxycarbonyl-(S)-2,4-diaminobutanoic acid (**2**): The reaction was adapted from literature [51]. *N*-tert-butoxycarbonyl-(*S*)-glutamine (**1**; Sigma; 5.0 g, 20.3 mmol) was dissolved in THF (48 mL) and water (12 mL). The solution was cooled in an ice bath, and (diacetoxyiodo)benzene (7.8 g, 24.2 mmol) was added. The reaction was monitored by TLC (4:1:1 n-butanol: acetic acid: water). After 6 hours, the organic portion was evaporated *in vacuo*. The aqueous portion was diluted with water, and extracted with ethyl acetate (3 x 30 mL). The aqueous portion was then partially evaporated, frozen, and lyophilised. The product *N*-tert-butoxycarbonyl-(S)-2,4-diaminobutanoic acid was obtained as an orange-yellow solid (3.1 g, 71%). IR (ν_max_, film): 3416, 2977, 2931, 1698, 1589, 1561, 1529, 1499, 1439, 1392, 1366, 1252, 1161, 1052, 1029, 949, 907, 868, 802. ^1^H NMR (400 MHz, D_2_O) δ: 3.86 (m, 1H, C*H*), 2.94 (t, *J* = 7.8 Hz, 2H, NH_2_C*H_2_*), 1.9 (m, 1H, C*H_2_*), 1.85 (m, 1H, C*H_2_*), 1.31 (s, 9H, C(C*H_3_*)_3_). ^13^C (100 MHz, D_2_O) δ: 28.3 ((CH_3_)_3_), 29.9 (Cβ), 39.1 (CH_2_N_3_), 57.1 (Cα), 80.9 ((CH_3_)_3_CCONH)**,** 157.9 ((CH_3_)_3_CCONH), 175.6 (COOH), LRMS (ESI) Calculated: [M-H] C_9_H_18_N_2_O_4_ (m/z): 218, Obtained: [M-H]^–^ : 217.12, [2M-H]^–^ : 435.26, [M+H]^+^: 219.16, Melting pt. = 196 ºC, Lit. [51] 190-191 ºC, [α]^20^_D_ = -8.6º (c = 1.0, H_2_O), Lit. [51] [α]^20^_D_ = -9.6 (c = 1.0, H_2_O).

2. *N*-tert-butoxycarbonyl-(*S*)-azidohomoalanine (**3**): Triflyl azide was prepared *in situ*, and the reaction was adapted from the literature [52]. Sodium azide (9.8 g, 150 mmol) was dissolved in a mixture of water (22 mL) and dichloromethane (39 mL). The contents were cooled in an ice bath, and triflic anhydride (5.1 mL) was added via syringe over 5 min. The contents were allowed to stir for 30 min, after which the layers were separated. The aqueous layer was extracted using dichloromethane (2 x 50 mL). The organic fractions were pooled, and washed twice with 5% aq. sodium carbonate (30 mL). The triflyl azide containing organic layer was then added to a solution of *N*-tert-butoxycarbonyl-(S)-2,4-diaminobutanoic acid (3.1 g, 14.2 mmol) in water (39 mL) and methanol (90 mL). To this, potassium carbonate (3.2 g) and copper sulphate pentahydrate (38.4 mg) were added. Stirring was continued for 18 h, after which the organic fraction was evaporated *in vacuo*. The aqueous portion was extracted with dichloromethane (2 x 50 mL), diluted with water, then acidified to pH 2.0 using 1.0 N hydrochloric acid. The acidified aqueous phase was then extracted with dichloromethane (3 x 50 mL). The organic phase was then washed with brine (2 x 80 mL), dried with magnesium sulphate, filtered, and evaporated. The product was obtained as orange oil (3.2 g, 92%). IR (ν_max_, film): 3319, 2980, 2935, 2100, 1690, 1513, 1455, 1399, 1369, 1236, 1189, 1154, 1093, 1057, 1029, 940, 853. ^1^H NMR (400 MHz, CDCl_3_) δ: 5.26 (br d, *J* = 8.8 Hz, 1H, NH), 4.41 (m, 1H, CH), 3.46 (t, *J* = 6.7 Hz, 2H, N_3_CH_2_), 2.11 (m, 1H, CH_2_), 1.98 (m, 1H, CH_2_), 1.46 (s, 9H, C(CH_3_)_3_). ^13^C (100 MHz, D_2_O) δ: 28.2 ((CH_3_)_3_), 31.4 (Cβ), 47.8 (CH_2_N_3_), 51.3 (Cα), 80.7 ((CH_3_)_3_CCONH), 155.7 ((CH_3_)_3_CCONH), 175.8 (COOH), LRMS (ESI) Calculated: [M-H] C_9_H_16_N_4_O_4_ (m/z) : 243, Obtained: [M-H]^–^ : 243.08, [2M-H]^–^ : 487.16, [α]^20^_D_ = +16.1º (c = 1.0, CHCl_3_), Lit. [54] [α]^20^_D_ = +19.0º (c = 1.0, CHCl_3_).

3. L-azidohomoalanine (**4**): The reaction was performed as reported in literature [53]. *N*-tert-butoxycarbonyl-(*S*)-azidohomoalanine (2.9 g, 11.88 mmol) was dissolved in 10 N HCl (30 mL), and the contents stirred at room temperature for 1 hour. The solution was then diluted with 170 mL water. 80 g of DOWEX50-WX8 resin was packed into a column, and conditioned by washing with 1N aq. ammonia (3 x 80 mL), water (600 mL), 1N HCl (250 mL), and water again until the eluate was at pH 7.0. The peptide solution was run through the column; the flow-through was collected and passed over the resin again. Aha was eluted from the column by washing with 500 mL of 1N aq. ammonia, and recovered as an off-white solid on evaporation. The compound was recrystallised from acetone/water to yield white crystals in 91% yield (1.54 g). IR (ν_max_, film): 3368, 3036, 2978, 2926, 2103, 1677, 1589, 1489, 1351, 1333, 1265, 1204, 1101, 854, 771. ^1^H NMR (400 MHz, D_2_O) δ: 3.99 (t, *J* = 6.32, 1H, CαH), 3.48 (T, *J* = 6.4, 2H, CH_2_N_3_), 2.07 (m, 2H, CβH_2_). ^13^C (100 MHz, D_2_O) δ: 29.6 (Cβ), 47.6 (CH_2_N_3_), 57.8 (Cα), 171.9 (COOH). LRMS (ESI) Calculated: [M+H] C_4_H_8_N_4_O_2_ (m/z): 144, Obtained: [M+H]^+^: 145.08, [M+HCl-H]^–^ : 179.06, [2M-H]^–^ : 287.11, Melting pt. 180.6 ˚C, [α]^20^_D_ = +6.60º (c = 0.50, H_2_O), Lit. [54] [α]^20^_D_ = +6.60º (c = 0.50, H_2_O).

**Half-lives**

**Figures 1B** and **3B** include a simple exponential decay curve

*i_t_*  _= (_*i_max_ –Plateau*_)_e^-λt^ *+ Plateau*

fitted to the data (using ‘Prism’; Graphpad) – but note that we do not know how many different kinetic populations there might be. The half-life (*t_1/2_*) of tagged peptides can then be calculated using *t_1/2_* = 0.693/λ where λ is the rate constant of exponential decay, *i_max_* is the maximum intensity (i.e., mean intensity after the pulse without a chase), and *i_t_* is the intensity at time ‘*t*’ (i.e., mean intensity at time ‘*t’* after initiating the chase). For labeling with Aha, heavy Met, and heavy Lys + Arg, *Plateau* values for the cytoplasm (and nucleus) were 03.5, 0.09, and 0.19 (and 3.3, 0.13, and 0.21) respectively, and half-lives were 33, 10, and 48 s (and 34, 10, and 32 s) respectively. *R^2^* values (the square of the Pearson product-moment correlation coefficient; high values indicate a good fit) for all exponential decay curves shown were >0.98, except for those given by heavy Lys + Arg (which gave values of 0.92 and 0.96 for cytoplasm and nucleus, respectively).

**Molecular cloning**

Three vectors were used (**Fig. S4**); all encode an SV40 *ori* (to permit replication in Cos7 cells; [55]) and a modified P^tight^ ‘Tet’ promoter inducible with doxycycline. In the base vector, the P^tight^ promoter drives expression of a protein containing an N-terminal mitochondrial signal sequence (mt), the rat CD2 epitope recognized by the OX34 antibody [56-57] and encoded by exons 2 and 3 of rat *Cd2*, a shortened intron 2 of *Cd2* (with intact splice donor and acceptor sites), a ribosome pause sequence (rps), a C-terminal EGFP, and a poly-adenylation signal. Vector 1 is as the base vector except that the EGFP sequence contains the segment encoding the EGFP fluorochrome deleted (to free the green channel for other use). Vector 2 is as the base vector except that a haemagglutinin (HA) tag is inserted within the intron (in-frame with exon 2 and upstream of the first intronic stop codon). Vector 3 is as Vector 2 but also bears a PTC 67 nucleotides upstream of the exon2-intron2 junction (a position that should induce NMD; [58]). CD2 protein and the HA tag were expressed in the expected manner by these vectors (not shown); here, we only analyze RNA expression.

The base vector was created as follows. pLdPDH [59] encodes the mitochondrial import sequence of human *PDH1α* inserted between the *Sac*I and *Pst*I sites in pEGFP-N1 (Clontech). The P^tight^ promoter from pTRE-Tight (Clontech) was inserted between the *Xho*I and *Eco*RI sites in pLdPDH. Two fragments of rat *Cd2* were amplified from total rat DNA (Bioline) using oligo pair 1 (which target chromosome 2, between positions 196,332,778-196,333,452; [60]) and oligo pair 3 nested with oligo pair 2 (which target chromosome 2 between positions 196,336,082-196,338,119; [60]). These two *Cd2* fragments of 676 and 2,057 bp (with *Nhe*I- and *Xba*I-compatible cohesive ends, respectively) were simultaneously inserted between the *Pst*I and *Bam*HI sites in pLdPDH (which now encodes P^tight^). The base vector was formed by removing from the doubly-modified pLdPDH the fragments containing P^tight^, the mitochondrial import sequence, and *Cd2*, and inserting the three between the *Xho*I and *Bam*HI sites in plasmid 0,*EGFP*,pA (a promoter-less form of pEGFP-N1; [30]). Vector 1 was constructed by deleting the EGFP chromophore (5ʹ-CTGACCTACGGCGTGCAG-3ʹ; 18 bp) from the base vector by site-directed mutagenesis (‘QuikChange Lightning kit’; Stratagene) using oligo pair 4. Vector 2 was constructed by inserting a sequence encoding the HA epitope (5ʹ-TACCCTTACGACGTTCCTGATTACGCT-3ʹ; 27 bp) in the base vector by site-directed mutagenesis using oligo pair 5. Unexpectedly, the insert proved to be 81 bp (i.e., 5ʹ-TACCCTTACGACGTTCCTGATTACGCTTAGCAAGTTGGGGGGACGGGTGCTATGTACCCTTACGACGTTCCTGATTACGCT-3ʹ) in which two HA encoding sequences were interrupted by a 27-bp sequence (note that the HA tag is not used here, so that this modification has no impact on the experiments discussed here). Vector 3 was constructed (by site-directed mutagenesis using oligo pair 6) by introducing a point mutation (5ʹ-TAT-3ʹ → 5ʹ-TAA-3ʹ) that creates an in-frame premature termination codon (PTC) in the *Cd2* exon in Vector 3. Correct construction of the vectors was confirmed by DNA sequencing after PCR with the following oligonucleotides. Presence of P^tight^ and *Cd2* fragments in the base vector was verified using oligos 7 and 8. Absence of the EGFP chromophore in Vector 1 was verified using oligo 9. Oligo 7 was also used to verify the presence of the HA-coding sequence in Vector 2 and the point mutation in Vector 3. Bacterial transformations were performed using One Shot TOP10 chemically-competent *Escherichia coli*. All PCRs (except for site-directed mutagenesis) were performed using PfuUltra II Fusion HS DNA Polymerase (Stratagene) at a re-annealing temperature of 58.6˚C (64˚C in case of oligo pair 3). All oligonucleotide DNA primers used (supplied by Sigma) are listed in **Table S1**.

**References for Supplementary Information**

1. Andruszkiewicz R, Rozkiewicz D (2004) An improved preparation of N 2‐tert‐butoxycarbonyl‐ and N 2‐benzyloxy‐carbonyl‐(S)‐2,4‐diaminobutanoic acids. Synth Commun 34: 1049–1056. doi: 10.1081/scc-120028636.
2. Lundquist JT, Pelletier JC (2001) Improved solid-phase peptide synthesis method utilizing α-azide-protected amino acids. Org Lett 3: 781–783. doi: 10.1021/ol0155485.
3. Link AJ, Vink MK, Tirrell DA (2007) Preparation of the functionalizable methionine surrogate azidohomoalanine via copper-catalyzed diazo transfer. Nat Protoc 2: 1879–1883. doi: 10.1038/nprot.2007.268.
4. Fernandez-Gonzalez M, Boutureira O, Bernardes GJL, Chalker JM, Young MA, et al. (2010) Site-selective chemoenzymatic construction of synthetic glycoproteins using endoglycosidases. Chem Science 1: 709–715. doi: 10.1039/c0sc00265h.
5. Mellon P, Parker V, Gluzman Y, Maniatis T (1981) Identification of DNA sequences required for transcription of the human α1-globin gene in a new SV40 host-vector system. Cell 27: 279–288. doi: 10.1016/0092-8674(81)90411-6.
6. Williams AF, Barclay AN, Clark SJ, Paterson DJ, Willis AC (1987) Similarities in sequences and cellular expression between rat CD2 and CD4 antigens. J Exp Med 165: 368–380. doi: 10.1084/jem.165.2.368.
7. Davis SJ, Davies EA, van der Merwe PA (1995) Mutational analysis of the epitopes recognized by anti-(rat CD2) and anti-(rat CD48) monoclonal antibodies. Biochem Soc Trans 23: 188–194. doi: 10.1042/bst0230188.
8. Brogna S, Wen J (2009) Nonsense-mediated mRNA decay (NMD) mechanisms. Nat Struct Mol Biol 16: 107–113. doi: 10.1038/nsmb.1550.
9. Margineantu DH, Brown RM, Brown GK, Marcus AH, Capaldi RA (2002) Heterogeneous distribution of pyruvate dehydrogenase in the matrix of mitochondria. Mitochondrion 1: 327–338. doi: 10.1016/s1567-7249(01)00033-2.
10. Gibbs RA, Weinstock GM, Metzker ML, Muzny DM, Sodergren EJ, et al. (2004) Genome sequence of the Brown Norway rat yields insights into mammalian evolution. Nature 428: 493–521. doi: 10.1038/nature02426.
11. Pestka S (1971) Inhibitors of ribosome functions. Annu Rev Microbiol 25: 487–562. doi: 10.1146/annurev.mi.25.100171.002415.
12. Katunin VI, Muth GW, Strobel SA, Wintermeyer W, Rodnina MV (2002) Important contribution to catalysis of peptide bond formation by a single ionizing group within the ribosome. Mol Cell 10: 339-346. doi: 10.1016/s1097-2765(02)00566-x.
13. Dieterich DC, Link AJ, Graumann J, Tirrell DA, Schuman EM (2006) Selective identification of newly synthesized proteins in mammalian cells using bioorthogonal noncanonical amino acid tagging (BONCAT). Proc Nat Acad Sci USA 103: 9482–9487. doi: 10.1073/pnas.0601637103.
14. Williamson AR, Schweet R (1965) Role of the genetic message in polyribosome function. J Mol Biol 11: 358–372. doi: 10.1016/s0022-2836(65)80063-8.
15. Stevens A, Maupin MK (1989) 5,6-Dichloro-1-beta-D-ribofuranosylbenzimidazole inhibits a HeLa protein kinase that phosphorylates an RNA polymerase II-derived peptide. Biochem Biophys Res Commun 159: 508–515. doi: 10.1016/0006-291x(89)90022-3.

**Legends to Supplementary Figures**

**Figure S1.** Aha incorporation; some controls.

HeLa or HUVECs were grown ± Met for 15 min, pulsed ± 2 mM Aha for 2 min, and in some cases chased with 0.2 mM Met (without Aha) at 37˚C or 4˚C for the times indicated; after fixation, Alexa555 was ‘clicked’ on to incorporated Aha, DNA counterstained with DAPI, images collected of Alexa 555 fluorescence using a wide-field microscope (typical views are shown), and intensities in the cytoplasm (*cyto*) and nucleus (*nuc*) measured. Bars: 10 µm.

**(A)** Aha incorporation by unstarved cells (using conditions exactly as in Fig. 1A, except that cells were not initially grown without Met). Panels (iii) and (iv) show the only results presented where background seen in the absence of Aha in the nucleus or cytoplasm was not subtracted. Alexa 555 fluorescence in the cytoplasm and nucleus is expressed (± SD) relative to the value found in the Aha-pulsed cytoplasm. *: *P* < 0.0001 (Student’s two-tailed *t* test, *n* = 20 cells).

**(i)** Omission of Aha yields background fluorescence.

**(ii)** Aha yields both cytoplasmic and nuclear signal (the latter is the strongest).

**(iii)** Signal is highest in nuclei, and nuclear signal is 3-fold higher than the (nuclear) background (– Aha).

**(iv)** Omission of Aha yields background fluorescence.

**(v)** Aha yields faint nuclear signal.

**(vi)** A faint – but nonetheless – significant signal (+ Aha) above background (– Aha) is seen in nuclei.

Some additional controls include:

(i) Anisomycin. In Figure 1A, 100 µg/ml anisomycin was added for 2 h to inhibit translation – conditions which are used routinely (e.g., [21]); we also use shorter times in critical experiments (e.g., 15 min in Fig. 1D).

(ii) Other inhibitors. Puromycin and cycloheximide give similar results to anisomycin, but neither inhibit Aha incorporation more quickly and/or to a greater extent than anisomycin, in accord with published data [61]. For example, after a 10-min pre-incubation in 2 mM puromycin, and a 20-s Aha pulse, the relative intensity of signal in nucleus and cytoplasm was reduced to 37 ± 2% and 28 ± 2%, respectively. It is unsurprising that inhibition is incomplete because the apparent *K_m_* of puromycin for the bacterial ribosome is ~3 mM [62]. Note also that cycloheximide inhibits Aha incorporation into peptides [63], and its effects on Aha incorporation are discussed in the brochure provided by Invitrogen (who supplied some of the Aha and reagents used for ‘click’ chemistry).

(iii) Non-specific binding to RNA and DNA. In an experiment like that in Figure S1D (involving a 5-s Aha pulse), treatment with 5U RiboShredder (Epicentre) – an RNase cocktail – plus 3% BSA (Sigma) in PBS after fixation and immediately prior to ‘clicking’ slightly increases the relative intensity in nucleus and cytoplasm to 189 ± 26% and 139 ± 22%; similarly, treatment (30 min; 37°C) with 4.7 units DNase I (Worthington) and 0.5 mM CaCl_2_ plus 3% BSA in PBS gave relative intensities of 177 ± 30% and 97 ± 17% (not shown). This is consistent with Aha not being attached inappropriately to RNA or DNA, and with the removal of nucleic acids increasing access of Alexa555 to Aha-labeled peptides during the ‘click’ reaction.

**(B)** Both nuclear and cytoplasmic signals disappear within minutes during a chase at 37°C. HeLa cells were starved of Met (15 min), pulsed for 2 min with 2 mM Aha, and chased for 0 or 5 min with 0.2 mM Met (without Aha) at 37˚C or 4˚C.

**(i-iii)** A 2-min Aha pulse labels both nucleus and cytoplasm, but essentially all signal disappears during a 5-min chase with Met at 37˚C – but not at 4˚C. Panel (ii) is the same as that in Figure 1Av.

**(iv)** After subtracting background, intensities (± SD) seen are expressed relative to the value found without a chase in cytoplasm. *: *P* < 0.0001 (Student’s two-tailed *t* test, *n* = 20 cells).

**(C)** Some longer-lived peptides are labeled with longer Aha pulses. HUVECs were pulsed (without prior Met starvation) for 2-60 min with 2 mM Aha, and chased for 0 or 5 min with 0.2 mM Met (without Aha) at 37˚C.

**(i)** After a 2-min Aha pulse (without a chase), the nucleus appears the brightest (signal in both compartments is low, as there was no prior Met starvation).

**(ii)** With a 2-min pulse and 5-min chase, essentially all signal disappears.

**(iii,iv)** As pulse length increases (and chase length remains constant), nuclear signal increases.

**(v)** After subtracting background, intensities (± SD) seen are expressed relative to the value found without a chase in the cytoplasm. *: *P* < 0.0004 (Student’s two-tailed *t* test, *n* = 20 cells). Some signal is seen in both nucleus and cytoplasm after the longest pulse, consistent with this signal marking the stable cellular proteome.

**(D)** During pulses of 5-15 s, Aha is incorporated into both nucleus and cytoplasm. HeLa cells were starved of Met for 30 min, and pulsed with 2 mM Aha for 0-15 s. After subtracting background, intensities (± SD) seen with Aha are expressed relative to the value found in the cytoplasm after a 15-s pulse. Signals in both compartments increase. *: *P* < 0.0001 (Student’s two-tailed *t* test, *n* = 20 cells). Adding 100 µg/ml anisomycin 15 min before a 5-s pulse reduces the relative intensity in nuclei by 54%, and in the cytoplasm by 85% (*P* < 0.0001, Student’s two-tailed *t* test, *n* = 20 cells).

**Figure S2.** Comparison of images obtained using wide-field and confocal microscopes.

**(A)** Comparison of images (Alexa 555 fluorescence) obtained using wide-field and confocal microscopes. Bar: 10 µm.

**(i,ii)** Labeling as in Figure 1Dii. The single confocal section through the centre of the nucleus yields a punctate signal.

**(iii,iv)** Labeling as in Figure 2B; to allow comparison, panel iv is the same as that in Figure 2B. Again, the single confocal section yields a more punctate signal. Puromycin: puro.

**Figure S3.** Puromycin incorporation: some controls. Bars: 10 µm.

Cells were pre-treated with 100 µg/ml cycloheximide (chx) for 15 min (to slow ribosomes), and pulsed ± 91 µM puromycin (puro) for various times; in some cases, cells were pretreated with 100 µg/ml anisomycin (aniso) for 2 h before fixation, and the pulse was followed by a chase at 37˚C or 4˚C. After fixation, puromycylated peptides were indirectly immuno-labeled with Cy3, DNA stained with DAPI, and images of Cy3 fluorescence collected using a confocal (all images shown are typical single confocal sections through the centre of nuclei) or wide-field microscope (used to determine relative intensities).

**(A)** The changing distributions of puromycylated peptides in HUVECs during pulse-chases.

**(i,ii)** After a 5-s pulse, puromycylated peptides are seen in foci in both cytoplasm and nucleus, with the latter being the brightest.

**(iii)** After a 5-s pulse followed by a 60-s chase at 37˚C, the peri-nuclear region now contains the brightest foci (presumably because puromycylated peptides made in both the nucleus and cytoplasm accumulate in the SER, and because some unincorporated puromycin remains within the cell during the chase to became incorporated).

**(iv)** After a 5-s pulse followed by a 60-s chase at 4˚C, the pattern remains more like that seen in (ii). In other words, the nuclear signal is brighter (which we attribute to the low temperature reducing translation and intra-cellular transport).

**(v)** After subtracting background, Cy3 intensities (± SD; *n* = 20 cells) are expressed relative to the value found in the cytoplasm after a 5-s pulse. Both nuclear and cytoplasmic signals increase after a 60-s chase at 37˚C; we attribute this to incomplete removal of all cellular puromycin on transfer from the puromycin-containing medium to the puromycin-free medium used for the chase. After a chase at 4˚C, the puromycin signal in both nucleus and cytoplasm increases only slightly. *: *P* < 0.0001 (Student’s two-tailed *t* test).

**(B)** Puromycin incorporation by HeLa. David et al*.* [5] obtained a diffuse nuclear signal (with some nucleolar labeling) and brighter perinuclear signal after performing analogous experiments to those in Figure 2 with two differences; they permeabilized cells before adding puromycin, and pulsed for 5 min. Therefore, we repeated our experiment (where we only permeabilize after fixation) using a 5-min pulse.

**(i)** With no puromycin pulse, background levels of signal are seen.

**(ii)** A 5-min pulse of puromycin yields significant peri-nuclear labeling; this is like David et al. [5]. Unlike David et al. [5], there is little nucleolar labeling (which could be due to their permeabilization allowing some mRNAs to enter nucleoli to be translated by the high concentration of ribosomes there).

**(iii)** Pretreatment with anisomycin reduces both cytoplasmic and nuclear signal.

**(iv)** After subtracting background, Cy3 intensities (± SD) are expressed relative to the value found in the untreated (– anisomycin) cytoplasm; anisomycin reduces significantly the relative intensity in both nucleus and cytoplasm (*: *P* < 0.0001, Student’s two-tailed *t* test, *n* = 20 cells).

**(C)** Effects of pre-treatment with cycloheximide (chx) on the distribution of puromycylated peptides in HeLa.

**(i,ii)** Without pre-treatment with cycloheximide, puromycylated peptides are seen in foci in both cytoplasm and nucleus, with the former yielding the brightest foci; with pre-treatment, the nucleus yields the brightest foci. This reversal is simply explained as follows. We assume a 5-s Aha pulse (where nuclear signal appears the brightest; Fig. 1Dii) gives the truest indication of the fraction of translation occurring in nuclei during a 5-s pulse. [This assumption seems appropriate as Aha-labeled peptides can only detach from the ribosome once the whole protein has been completed, whereas incomplete proteins that become end-labeled with puromycin are known to detach rapidly [64] and become concentrated at exit sites in the SER [5], [27].] In the absence of cycloheximide, some puromycylated peptides made in both the nucleus and cytoplasm become concentrated in the SER; then, some labeled peptides will move from nucleus to cytoplasm to increase the cytoplasmic signal. However, in the presence of cycloheximide, ribosomes are slowed equally in both compartments, and more puromycylated peptides remain at the ribosome. As a result, the bias towards the cytoplasm is minimized (and the nucleus now appears brighter than the cytoplasm).

**(iii)** After subtracting background, Cy3 intensities in wide-field images are expressed relative to the value found in the cytoplasm after a 5-s puromycin pulse without pre-incubation in cycloheximide. The nuclear and cytoplasmic signal is reduced by cycloheximide (*: *P* < 0.0001, Student’s two-tailed *t* test, *n* = 20 cells).

**(D)** Pre-treating HeLa cells with a transcriptional inhibitor slightly reduces puromycylation (5-s pulse) in the nucleus.

**(i)** Confocal sections through the center of nuclei. (i) Puromycylated peptides are seen in foci in both cytoplasm and nucleus; the latter yields the brightest foci. (ii) 100 µg/ml anisomycin added 30 min before puromycin marginally reduces nuclear signal. (iii) 100 µM DRB (5,6-dichloro-1-β-ᴅ-ribofuranosylbenzimidazole; [65]), added 30 min before puromycin also slightly reduces nuclear signal, without affecting the cytoplasmic one.

**(iv)** Quantitation using wide-field images. After subtracting background, intensities (± SD) seen with puromycin are expressed relative to the value found in the untreated (–anisomycin/–DRB) cytoplasm. Due to integration over the larger area, most signal is again seen in the cytoplasm (despite nuclear foci being the brightest). Anisomycin appears to have less effect on the cytoplasm; we attribute this to cycloheximide reducing both nuclear and cytoplasmic translation by 40-50% (Fig. S3Ciii), coupled with labeled peptides made in the cytoplasm remaining in the cytoplasm whereas those made in the nucleus are transported to the cytoplasm. Adding DRB slightly reduces nuclear signal (without effect on the cytoplasmic one), consistent with some close coupling of nuclear translation with transcription. *: *P* < 0.0001 (Student’s two-tailed *t* test, *n* = 20 cells). If translation occurs only in the cytoplasm, the reduction in nuclear signal can only be explained by splicing, mRNA export, cytoplasmic translation, and (an extraordinary) import of puromycylated peptides into nuclei all occurring in 5 s – which seems unlikely.

**Figure S4.** CD2-EGFP expression constructs. Each construct encodes an SV40 *ori* (to permit replication) and a modified ‘Tet’ promoter inducible with doxycycline. In the base vector, the ‘Tet’ promoter drives expression of a protein containing an N-terminal mitochondrial signal sequence (mt), the rat CD2 epitope recognized by the OX34 antibody, a shortened intron 2 of *Cd2* (with intact splice donor and acceptor sites), a ribosome pause sequence (rps) which should slow a translating ribosome, and a C-terminal EGFP. Vector 1 encodes an EGFP sequence with the segment encoding the fluorochrome deleted (nfEGFP, to free the green channel for other use). Vector 2 encodes a haemagglutinin (HA) tag (not used in this study) inserted within the intron upstream of the first stop codon. Vector 3 also bears a PTC, 67 nucleotides upstream of the intron-exon junction (a position that should induce NMD; [58]).
